# M1-P15 as a cortical marker for transcallosal inhibition: A preregistered TMS-EEG study

**DOI:** 10.3389/fnhum.2022.937515

**Published:** 2022-09-16

**Authors:** Agnese Zazio, Guido Barchiesi, Clarissa Ferrari, Eleonora Marcantoni, Marta Bortoletto

**Affiliations:** ^1^Neurophysiology Lab, IRCCS Istituto Centro San Giovanni di Dio Fatebenefratelli, Brescia, Italy; ^2^Cognition in Action (CIA) Unit - PHILAB, Department of Philosophy, University of Milan, Milan, Italy; ^3^Statistics Unit, IRCCS Istituto Centro San Giovanni di Dio Fatebenefratelli, Brescia, Italy

**Keywords:** TEPs, effective connectivity, interhemispheric inhibition, motor system, ipsilateral silent period, bimanual coordination

## Abstract

In a recently published study combining transcranial magnetic stimulation and electroencephalography (TMS-EEG), an early component of TMS-evoked potentials (TEPs), i.e., M1-P15, was proposed as a measure of transcallosal inhibition between motor cortices. Given that early TEPs are known to be highly variable, further evidence is needed before M1-P15 can be considered a reliable index of effective connectivity. Here, we conceived a new preregistered TMS-EEG study with two aims. The first aim was validating the M1-P15 as a cortical index of transcallosal inhibition by replicating previous findings on its relationship with the ipsilateral silent period (iSP) and with performance in bimanual coordination. The second aim was inducing a task-dependent modulation of transcallosal inhibition. A new sample of 32 healthy right-handed participants underwent behavioral motor tasks and TMS-EEG recording, in which left and right M1 were stimulated both during bimanual tasks and during an iSP paradigm. Hypotheses and methods were preregistered before data collection. Results show a replication of our previous findings on the positive relationship between M1-P15 amplitude and the iSP normalized area. Differently, the relationship between M1-P15 latency and bimanual coordination was not confirmed. Finally, M1-P15 amplitude was modulated by the characteristics of the bimanual task the participants were performing, and not by the contralateral hand activity during the iSP paradigm. In sum, the present results corroborate our previous findings in validating the M1-P15 as a cortical marker of transcallosal inhibition and provide novel evidence of its task-dependent modulation. Importantly, we demonstrate the feasibility of preregistration in the TMS-EEG field to increase methodological rigor and transparency.

## Introduction

The combination of transcranial magnetic stimulation and electroencephalography (TMS-EEG) provides a unique perspective on effective connectivity (Miniussi and Thut, 2010; Bortoletto et al., 2015), defined as the description of causal relationships between brain areas (Friston et al., 2013). Since pioneering studies, it has been shown that TMS-evoked potentials (TEPs) represent the propagation of cortical responses from the stimulated area to the connected ones (Ilmoniemi et al., 1997; Bonato et al., 2006), conveying state-dependent information (Massimini et al., 2005; Morishima et al., 2009). Moreover, recent research suggests that TMS signal propagates mainly along structural connections of the stimulated networks (Momi et al., 2021a,b; Esposito et al., 2022).

TEPs have been exploited to study interhemispheric communication, and specifically transcallosal connections between motor cortices. A first measure of interhemispheric signal propagation has been described by Voineskos et al. (2010), consisting of the ratio between TEPs recorded from contralateral and ipsilateral EEG channels over a time window of about a hundred of ms after the TMS pulse, which has been shown to have an inverse relationship with the microstructural integrity of the corpus callosum. This approach has been proven to be informative as a global measure of interhemispheric signal propagation in subsequent studies, both on healthy participants and in clinical populations (Jarczok et al., 2016; Casula et al., 2020, 2021; Hui et al., 2020). However, the transcallosal conduction delay (TCD), defined as the timing of interhemispheric connectivity along the fibers of the corpus callosum, is much faster and occurs within 20 ms in the motor system (Koganemaru et al., 2009; Caminiti et al., 2013). In this context, preliminary TMS-EEG evidence had suggested that TEPs can provide information on TCD, showing that after primary motor cortex (M1) stimulation the signal is transferred to the contralateral motor areas in the first tens of ms (Ilmoniemi et al., 1997; Zazio et al., 2021). Therefore, we have recently proposed to study TCD by measuring early TEPs occurring within 20 ms after the TMS pulse, exploiting the excellent temporal resolution of EEG (Bortoletto et al., 2021).

In our recent study (Bortoletto et al., 2021), we combined TMS-EEG with diffusion tensor imaging (DTI), which provides microstructural information on callosal integrity, and with the ipsilateral silent period (iSP), a well-known peripheral measure of interhemispheric inhibition obtained from TMS and electromyography (Meyer et al., 1995). Our results provided first evidence of M1-P15, a positive component occurring ~15 ms after M1 stimulation, as a TEP-derived measure of transcallosal inhibition between motor cortices. Indeed, the latency of M1-P15 was predicted by DTI structural connectivity, such that the higher the diffusivity along the fibers of the body of the corpus callosum, the shorter the latency of M1-P15. This first evidence suggested that M1-P15 latency may be considered a measure of TCD. Moreover, our findings indicated that the amplitude of M1-P15 reflects the strength of the transcallosal inhibition, as shown by a positive relationship with the magnitude of the iSP. Finally, the TCD as indexed by M1-P15 latency was associated with bimanual coordination performance, such that shorter left-to-right together with longer right-to-left TCD was associated with better temporal performance in bimanual finger opposition movements.

Considering the technical challenges inherent in the study of early EEG responses to TMS (Veniero et al., 2009; Ilmoniemi et al., 2015), and more generally the acknowledged reproducibility crisis in neuroscience (Button et al., 2013; Poldrack et al., 2017; Pavlov et al., 2021), further evidence is needed to strengthen the validation of the M1-P15 as measure of transcallosal connectivity. Similarly, the relationship between M1-P15 and bimanual coordination requires further investigation: First, despite a relationship between M1-P15 latency and behavioral performance was expected based on previous theories (Ringo et al., 1994), the interaction with the direction of information flow between hemispheres was not. Second, in the original study the M1-P15 and the bimanual performance were recorded separately and under different conditions, leaving an open question on whether the M1-P15 could be recorded also during the bimanual task and whether its latency was predictive of bimanual performance. Finally, another unexplored topic regards the possibility of modulating transcallosal inhibition, which would represent evidence supporting the physiological nature of M1-P15 and thus a further validation of this early TEP component. Therefore, we designed a conceptual replication study (Zwaan et al., 2018) in which we aimed at replicating evidence for validating the M1-P15 as physiological index of interhemispheric connectivity and its role in bimanual coordination, under experimental circumstances that could overcome some limitations of the original study.

Based on previous findings, we designed a new TMS-EEG study with the following main aims and hypotheses: (i) Validating M1-P15 as a measure of transcallosal inhibition: we expect to replicate the positive relationship between M1-P15 amplitude and the magnitude of iSP; (ii) Evaluating the behavioral relevance of M1-P15: we expect to replicate the relationships between M1-P15 latency and bimanual coordination during the sequential thumb-to-finger opposition task; if the replication was successful, we aimed at exploring the potential cost of TCD asymmetry on unimanual performance; (iii) Validating the M1-P15 through the modulation of the transcallosal inhibition: by manipulating the activity of the hand contralateral to the stimulation, we expect to modulate indexes of transcallosal inhibition, i.e., the iSP and the M1-P15.

Our hypotheses, as well as the methods and the analyses we planned to run, were preregistered on Open Science Framework (OSF) before data collection (https://osf.io/pg78j/), aiming at increasing the transparency of the research process, avoiding the risks of undisclosed analytic flexibility and providing an unbiased picture of the results, overall contributing in improving scientific replicability (Poldrack et al., 2017; Paul et al., 2021).

## Materials and methods

### Participants

Thirty-two right-handed healthy volunteers were enrolled in the study after giving written informed consent (19 women; mean age ± SD: 29.7 ± 8.6 years, range: 19–48 years; see Supplementary material for sample size estimation). Participants who took part in the study by Bortoletto et al. (2021) were not enrolled in the present study. They had no history of neurological disorders nor contraindications to TMS (Rossi et al., 2020). The study was performed in accordance with the ethical standards of the Declaration of Helsinki and approved by the Ethical Committee of the IRCCS Istituto Centro San Giovanni di Dio Fatebenefratelli (Brescia, 54-2019). One participant did not complete all the experimental blocks, leaving 31 subjects for the off-line analyses. In the off-line analyses, two additional participants were excluded due to the presence of residual artifacts, one in TMS-EEG and one in the iSP preprocessing, respectively.

### Design and procedure

**Figure 1:**
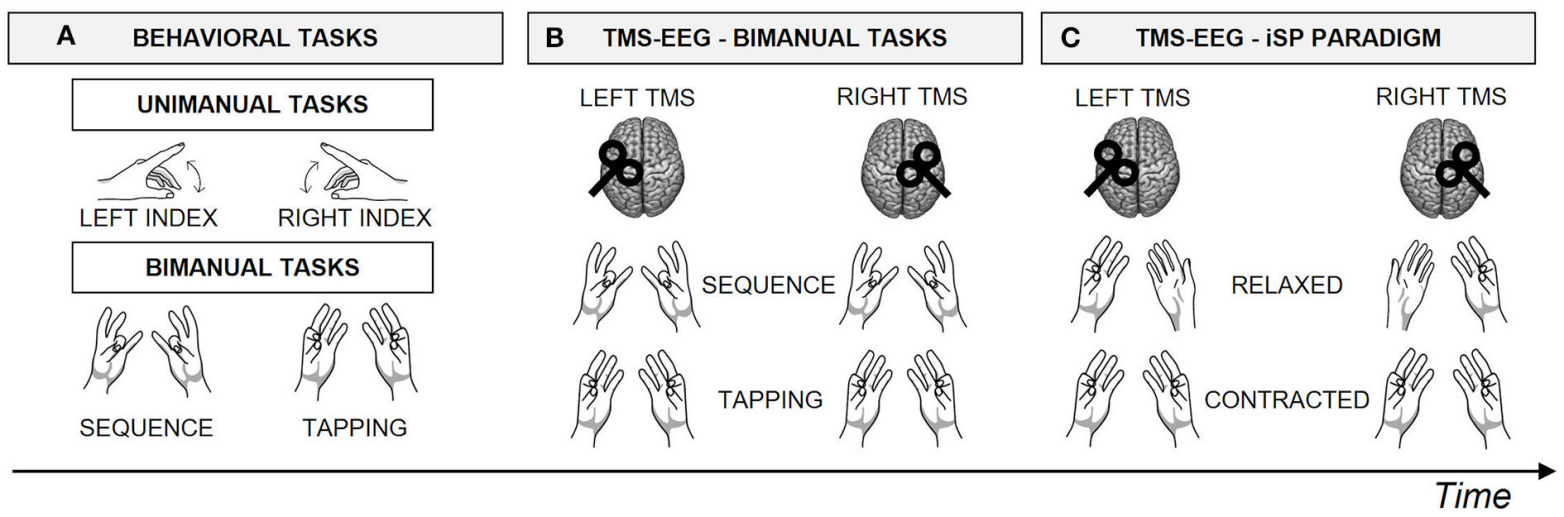
Experimental design. Schematic representation of the within-subject single-session design experiment, for a total duration of ~2.5 h. Block order was counterbalanced among participants using a Latin square design. **(A)** First, participants performed the behavioral tasks, including a unimanual task performed with the left and the right hand, followed by two bimanual tasks, i.e., *Sequence* and *Tapping*. **(B)** Then, participants performed the *Sequence* and the *Tapping* tasks during TMS-EEG recording, after stimulation of left and right M1, in separate blocks. **(C)** Finally, participants performed an iSP paradigm during TMS-EEG recording after stimulation of left and right M1, in separate blocks. Specifically, in the iSP paradigm the APB muscle of the hand ipsilateral to TMS was always *contracted*, while the contralateral APB was *Relaxed* or *Contracted*, in separate blocks.

Participants have been involved in a within-subject single-session design experiment (Figure 1), comfortably seated in front of a computer monitor with their forearms leaning on a desk.

#### Behavioral tasks

Behavioral tasks (Figure 1A) consisted of an unimanual task and two bimanual coordination tasks. Participants wore disposable gloves, and a conductive sensor was applied on each of the participants' fingertips with a double sided tape. In this way, sensors could be adjusted according to hand size and shape.

In the unimanual motor task, participants were asked to tap as fast as possible with their index finger on a sensor placed on the desk within a time interval of 10 s, while fixating a cross in the center of the screen. The ‘go' signal was represented by the onset of a white noise, delivered through earphones. In different blocks, they were asked to use their right and left index finger, respectively, performing 2 runs for each hand; hands order was counterbalanced across participants.

The bimanual coordination tasks involved metronome-paced in-phase movements at 2 Hz: a repetitive mirror-symmetrical thumb-to-index opposition task (*Tapping*) and a sequential mirror-symmetrical thumb-to-finger opposition task (*Sequence*), as in Bonzano et al. (2008) and Bortoletto et al. (2021). In the *Sequence* condition, participants were asked to oppose their thumb to the other fingers, in the following order: index, middle, ring and little finger. Participants performed 3 blocks for each bimanual task; each block lasted about 1 min. The metronome sound was presented through the earphones, overlapping with a white noise. The white noise was added to make the bimanual tasks comparable in the behavioral tasks and in the TMS-EEG recording.

#### TMS-EEG recording

Single biphasic TMS pulses were delivered over left (LTMS) and right (RTMS) M1 using a Magstim Rapid^2^ stimulator connected to an Alpha B.I. Coil Range 70 mm (Magstim Company, Whitland, UK), while EEG was continuously recorded with a TMS-compatible system (g.HIamp, g.tec medical engineering GmbH, Schiedlberg, Austria). The motor hotspot for APB muscle was localized as the scalp site eliciting the highest and most reliable motor-evoked potentials (MEPs) with the same TMS intensity. Coil orientation was kept ~45° from the midline, inducing an anterior-to-posterior and posterior-to-anterior (AP-PA) current direction in the brain. Then, the resting motor threshold (rMT) was estimated using the maximum-likelihood threshold hunting algorithm (Awiszus, 2003, 2011), a variant of the best parameter estimation by sequential testing (best PEST) procedure (Pentland, 1980). This procedure was performed for each hemisphere. TMS intensity was set at 110% of the average rMT (expressed as percentage of the maximal stimulator output - MSO) estimated for the left and the right hemisphere, as in our previous study (Bortoletto et al., 2021) (mean rMT ± SE: LTMS, 62 ± 2.1%; RTMS: 62.8 ± 2.0%). The charge delay was set at 350 ms and coil position was monitored with a neuronavigation system (Softaxic 3.4.0; EMS, Bologna, Italy). To attenuate the contamination of TEPs with sensory artifacts, a thin layer of foam was applied on the coil, and participants wore noise-canceling earphones playing white noise. Before starting the experiment, the volume was individually adjusted to mask the TMS click, as follows. The white noise was first played at a low level volume, and a few trial pulses at the 110% of the rMT were delivered close to the participants' head; participants were instructed to report whether they could hear the TMS click. If so, the volume was increased by 10% steps until it successfully masked the click or in case it reached hearing discomfort. At the end of the experiment, participants filled a questionnaire regarding the sensations associated with TMS. EEG was recorded by using 74 passive Ag/AgCl electrodes with 10/10 international system; the reference was placed on FPz and the ground on the nose (EasyCap, BrainProducts GmbH, Munich, Germany). Skin/electrode impedance was kept below 5 kΩ. EMG was recorded with a bipolar belly-tendon montage on left and right APB (Ag/AgCl pre-gelled surface electrodes, Friendship Medical, Xi'an, China). Sampling rate was set at 9600 Hz and no filters were applied in recording.

During the bimanual tasks (Figure 1B), the experimental blocks were the same as in the behavioral tasks (i.e., 3 blocks for *Tapping* and 3 blocks for *Sequence*), and they were performed twice, once during LTMS and once during RTMS (block order counterbalanced among participants). TMS pulses were randomly delivered in the intervals between sounds, based on the results of a pilot study showing that M1-P15 could be recorded at any time of TMS delivery during bimanual movements (see Pilot Study in Supplementary material). Therefore, TMS pulses occurred at 30 different time intervals (between 0 and 500 ms) after the metronome sound, with an inter-stimulus interval between TMS pulses of at least 1 s, as in the original study; 90 pulses were delivered for each condition. The jittered TMS pulse interval was chosen to record M1-P15 component without the time-locked contamination of auditory evoked potentials. Moreover, the presence of the metronome sounds both in the *Tapping* and in the *Sequence* rule out possible confounds related to the auditory-evoked potentials in the comparison between the two conditions.

During the iSP paradigm (Figure 1C), participants were asked to keep their ipsilateral APB muscle *contracted*, while the contralateral APB was either at rest (*Relaxed*) or *contracted* at the same level of strength (*Contracted*), during LTMS and RTMS in separate blocks in counterbalanced order. To this aim, at the end of the bimanual tasks during TMS-EEG recording, sensors were removed from the participants' fingers. Instead, a pressure sensor was applied on the second phalanx of their left and right index finger. Participants were asked to press on the sensor with their thumb, thus inducing a contraction of APB muscle. First, we estimated the maximal strength: participants were asked to press as strong as possible with their thumb on the sensor for 10 s, with the left and the right hand in separate runs. For each hand, the pressure values were averaged over a sliding window of 500 ms, and the 500 ms bin with the highest average value in the 10 s interval was selected as the maximum strength. Then, the main blocks started: in the *Contracted* conditions participants were asked to press on the sensors with both hands, while in the *Relaxed* conditions they had to press on the sensor only with their hand ipsilateral to TMS, keeping the contralateral hand relaxed. Participants were asked to press between 40 and 60% of their maximal strength. Every time the pressure level fell out of the required range, a visual feedback appeared on the screen indicating the actual pressure compared to the target range. Once the pressure level was reached, visual feedback disappeared and only the fixation cross remained in the center of the screen, to avoid eye movements during TMS-EEG recording. TMS was delivered only when the pressure on the sensors was within the target range, for a total of 90 pulses per condition. A break of ~30 s was provided in the middle of the blocks (i.e., after ~1.5 min). Moreover, participants were instructed to relax their hands anytime they needed an additional break; in this way, the TMS delivery was automatically interrupted until the pressure level had reached again the requested level.

### Analysis

#### Behavioral performance

Performance in the bimanual tasks was evaluated as the inter-hand interval (i.e., unsigned time difference in ms) between the onset of finger tap with the left hand and onset of the corresponding finger tap with the right hand (inter-hand interval values longer than 150 ms were excluded, as well as the ones exceeding 2 SD within each subject; Bortoletto et al., 2021). Since we calculated the absolute value of inter-hand interval, data were log-transformed to avoid skewness.

#### TEPs

TEPs data analysis was performed in MATLAB R2020b (The Mathworks, Natick, MA, USA) with custom scripts using EEGLAB v.2020.0 (Delorme and Makeig, 2004) and FieldTrip (Oostenveld et al., 2011) functions. If not otherwise specified, default parameters for EEGLAB and FieldTrip function were used. For each participant, the first pre-processing steps merged in the same dataset the two conditions of interest with the same stimulation site: for example, the conditions LTMS-*Contracted* and LTMS-*Relaxed* were analyzed as one dataset (the same for RTMS-*Contracted* and RTMS-*Relaxed*, LTMS-*Tapping* and LTMS-*Sequence*, RTMS-*Tapping* and RTMS-*Sequence*). This approach minimized the risk that differences between conditions that we compared could arise from dissimilarities in the preprocessing steps. The effect of the main steps of the preprocessing on the TMS-EEG data of a single participant are shown in Supplementary Figure S3.

**Figure 2:**
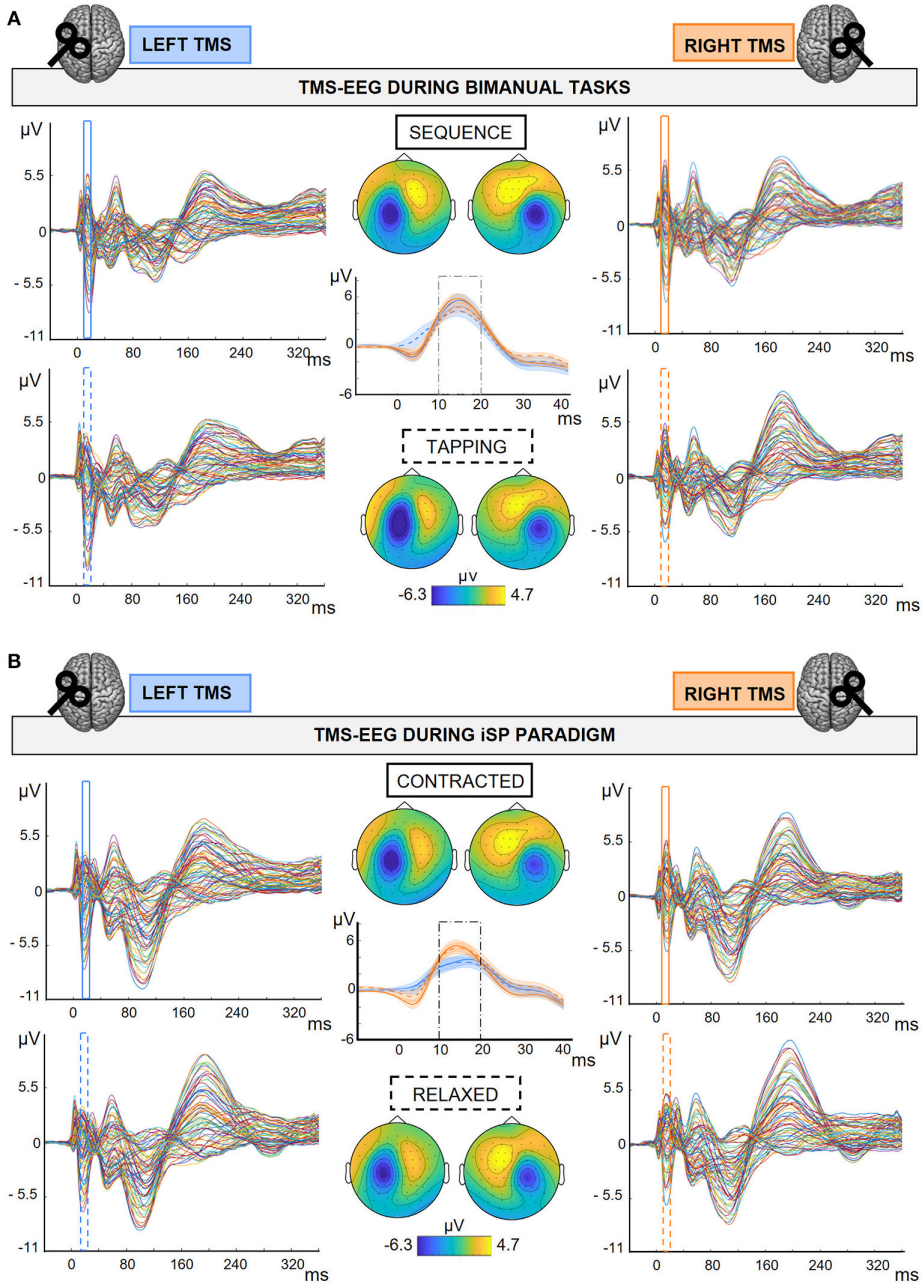
TMS-EEG output in the different experimental conditions. Grand average (*N* = 30) of TEPs and M1-P15 topographies obtained during the bimanual tasks **(A)** and the iSP paradigm **(B)**. M1-P15 topographies were obtained by averaging over time between 10 and 20 ms after the TMS pulse; amplitude range as indicated in colorbars. Central panels show M1-P15 on the average of F4-FC4 channels after LTMS (blue), and F3-FC3 after RTMS (orange); SE in shaded error bars. **(A)** 2 × 2 Hemisphere (LTMS, RTMS) X Task (*Sequence, Tapping*) design; Top row and continuous traces*:* TMS-EEG data during *Sequence;* bottom row and dashed traces*:* TMS-EEG data during *Tapping*. **(B)** 2 × 2 Hemisphere (LTMS, RTMS) X Contraction (*Contracted, Relaxed*) design; Top row and continuous traces*:* TMS-EEG data during *Contracted;* bottom row and dashed traces*:* TMS-EEG data during *Relaxed*.

Continuous TMS-EEG data was interpolated for 3 ms around the TMS trigger, high-pass filtered at 1 Hz (windowed sinc FIR filter using EEGLAB function ‘pop_eegfiltnew', order 31681), downsampled at 4800 Hz and epoched from 200 ms before to 500 ms after the TMS pulse. Then, the source-estimate-utilizing noise-discarding (SOUND) algorithm (Mutanen et al., 2018); spherical 3-layer model, regularization parameter: λ = 0.01) was applied to discard noise measurement, and a first artifact rejection was performed to discard highly artifactual trials based on visual inspection. After SOUND, we run an independent component analysis (ICA) for ocular artifact correction using the EEGLAB function ‘pop_runica' (infomax algorithm, 73 channels included, 73 ICA components computed; components relative to horizontal and vertical ocular movements are visually inspected and discarded). Then, we applied the signal-space projection and source-informed reconstruction (SSP-SIR) algorithm (Mutanen et al., 2016) for TMS-evoked muscular artifact removal (correction in the first 50 ms after TMS pulse). Principal components were visually inspected and discarded if they represented a high-frequency signal time-locked the TMS pulse; this step has been performed by two independent researchers (on average, 1.2 ± 0.09 SSP-SIR components were removed in a range between 0 and 5). Finally, after a low-pass filter at 70 Hz (IIR Butterworth filter, order 4, using the EEGLAB function ‘pop_basicfilter'), data was visually inspected as a final check to discard trials with residual artifacts, off-line re-referenced to the average of the left and the right mastoid, epoched from−100 to 400 ms, baseline corrected in the 100 ms preceding the TMS, and averaged according to the experimental condition (Figure 2). On average, 97.5% trials per condition were kept after the preprocessing. No channels were removed. The analysis steps followed the steps of the preregistered pipeline (https://osf.io/pg78j/), except for the high-pass filter on the continuous TMS-EEG data, which was modified into 1 Hz instead of 0.1 Hz for computational demands. Although in the present work we were not interested in slow potentials, we preliminary checked on the Pilot data that high-pass filtered TEPs at 1 or 0.1 Hz were qualitatively comparable (Supplementary Figure S2).

The M1-P15 identification was performed as follows. For each subject we first averaged over all conditions following the stimulation of the same hemisphere to increase the signal-to-noise ratio (i.e., *Tapping, Sequence, Contracted* and *Relaxed* during LTMS, and *Tapping, Sequence, Contracted* and *Relaxed* during RTMS). From the grand-average of all participants we identified the channels contralateral to TMS with the highest amplitude in the time window between 7 and 25 ms: F4-FC4 for LTMS, F3-FC3 for RTMS. Then, we identified the maximum on the pooled signal of the two channels in the time window between 7 and 25 ms; in case more than one peak was present or no peak could be detected, we manually restricted the time-window to match the M1-P15 topography (i.e., contralateral positivity over frontocentral electrodes). Then, we divided the data into the different experimental conditions, and the M1-P15 peak was automatically detected within 10 ms around the individual peak identified previously. M1-P15 amplitude was calculated by averaging over 10 ms around the peak. Overall, this procedure allowed us to perform a semi-automatic peak detection, avoiding subjective bias between the experimental conditions that we aim to compare.

#### iSP

The first preprocessing steps for the EMG trace ipsilateral to TMS were the same as for TMS-EEG data (i.e., interpolation, high-pass filter, downsample and epoching). Then, EMG was band-pass filtered between 100 and 1000 Hz (default FieldTrip filters) and rectified. For each participant, for each trial we checked whether the signal was lower than the mean of prestimulus baseline (calculated between 150 and 50 ms before the TMS pulse) for at least 10 ms in the time window between 25 and 75 ms after the TMS pulse; trials which did not satisfy this criterion were discarded. On average, 58.5 trials (64.9%) were considered in the average of each condition (range: 26–87 trials). Then, for each participant and condition we averaged over trials, and calculated the iSP with the same parameters as the ones applied on single trials. One subject was excluded in this step because no iSP was detected, leaving 29 participants for the statistical analyses involving the iSP. Finally, we calculated the normalized iSP area using the following formula [(area of the rectangle defined as mean EMG x iSP duration)–(area underneath the iSP)]. Then, the normalized iSP area was defined as the ratio between the iSP area and the area underneath EMG from - 150 to 50 ms preceding TMS (Trompetto et al., 2004; Bortoletto et al., 2021). Even if the number of trials considered for iSP calculation are consistent with the iSP literature, they represent only a subset of the trials presented and that were considered for M1-P15 calculation. To check whether the relationship between M1-P15 amplitude and iSP normalized area was maintained even when considering a comparable number of trials for the iSP estimation, in a control analysis we calculated the iSP by using 5 ms as minimum duration, both on single trials and on the average, leaving on average 86.4 trials for each condition (96%, range 75–90).

### Statistical analysis

If not otherwise specified, the statistical analyses followed what planned in the preregistration (https://osf.io/pg78j/). Details on the statistical models and results can be found in Supplementary Tables S1–S3.

(i) To validate M1-P15 as a measure of interhemispheric inhibition, we run a linear mixed model (LMM) testing whether M1-P15 amplitude predicted the iSP normalized area. We also performed two explorative (i.e., not defined in the preregistration) control analyses to exclude that the relationship between M1-P15 amplitude and the iSP normalized area could be explained by a common factor, namely the TMS intensity: For this we ran two LMMs testing whether rMT was predictive of M1-P15 amplitude and of iSP normalized area. LMMs were chosen for consistency with our previous work (Bortoletto et al., 2021) and to allow to consider multiple measures for each participants, thus increasing statistical power.
(ii) LMMs were also applied to assess the behavioral relevance of M1-P15. Bimanual coordination performance in the *Sequence* task was set as dependent variable, considering the inter-hand interval of each Tap of the bimanual task. For each subject, we considered an equal number of taps, by selecting the first *N* taps (where *N* is the number of taps of the participant with the minimum number of taps; *N* = 265). In separate models, we first considered as predictor the M1-P15 latency measured during the iSP paradigm and during the bimanual *Sequence* task. Separate LMMs tested M1-P15 latency during LTMS, RTMS and the ratio between the two (LTMS/RTMS) as predictors (considering the mean value of *Contracted* and *Relaxed* for the iSP paradigm). Since no significant relationships were observed between M1-P15 and bimanual performance in the *Sequence* (see Results), the preregistered analyses on the relationships between M1-P15 and bimanual performance in the *Tapping*, as well as between M1-P15 and unimanual performance, were not performed. We also ran additional control analyses on the behavioral tasks. We compared the bimanual performance between *Sequence* and *Tapping* by means of a two-tailed *t*-test for dependent samples, and we checked for an effect of time by comparing the bimanual performance in the three blocks, as indexed by the inter-hand interval measured during *Sequence*, by means of one-way repeated-measures analysis of variance (rm-ANOVA) with the 3-level factor Block. We also explored possible modulations of M1-P15 latency, both during the bimanual tasks (2 × 2 rm-ANOVAs with factors Hemisphere and Task) and during the iSP paradigm (2 × 2 rm-ANOVAs with factors Hemisphere and Contraction). ANOVA were performed because we were interested in testing differences between means taking into consideration the repeated-measure design.
(iii) To assess the modulation of the interhemispheric inhibition, we first tested the effects of contraction levels of the hand contralateral to the stimulation. We performed multivariate analysis of variance for repeated measures (rm-MANOVA) on M1-P15 amplitude and iSP normalized area, with Contraction (*Contracted, Relaxed*) and Hemisphere (LTMS, RTMS) as predictors. This statistical model was chosen because we were interested in the effect of Contraction on the two dependent variables. Considering the 2 × 2 design of the rm-MANOVA, a semi-parametric model was performed (modified ANOVA-type statistic—MATS). *Post-hoc* analyses were performed by separate 2 × 2 rm-ANOVAs on M1-P15 amplitude and the normalized iSP area, respectively. As additional control analysis, the rm-ANOVA was performed to test the effect of Contraction on the (non-normalized) iSP area, as this was the quantification method used in the original study by Giovannelli et al. (2009). Furthermore, we explored whether M1-P15 amplitude was modulated by the bimanual task performed by running a 2 × 2 rm-ANOVA with factors Hemisphere (LTMS, RTMS) and Task (*Sequence, Tapping*). Finally, we compared the rMT for the left and the right hemisphere by means of a two-tailed dependent sample *t*-test.

Statistical significance was set at *p* < 0.05. LMMs and rm-MANOVA were run in R software v. 4.0.0 (R Core Team, 2020) using the lme4 and the MANOVA.RM package, respectively, and remaining statistical analyses were performed using jamovi v. 1.6.15 (The jamovi project, 2021). If not otherwise specified, mean ± SE is reported in parentheses.

## Results

M1-P15 was visible with a comparable topography for all conditions of the bimanual tasks and of the iSP paradigm, for both left M1 and right M1 stimulation (Figure 2; individual plots can be found in Supplementary Figures S4–S7).

**Figure 3:**
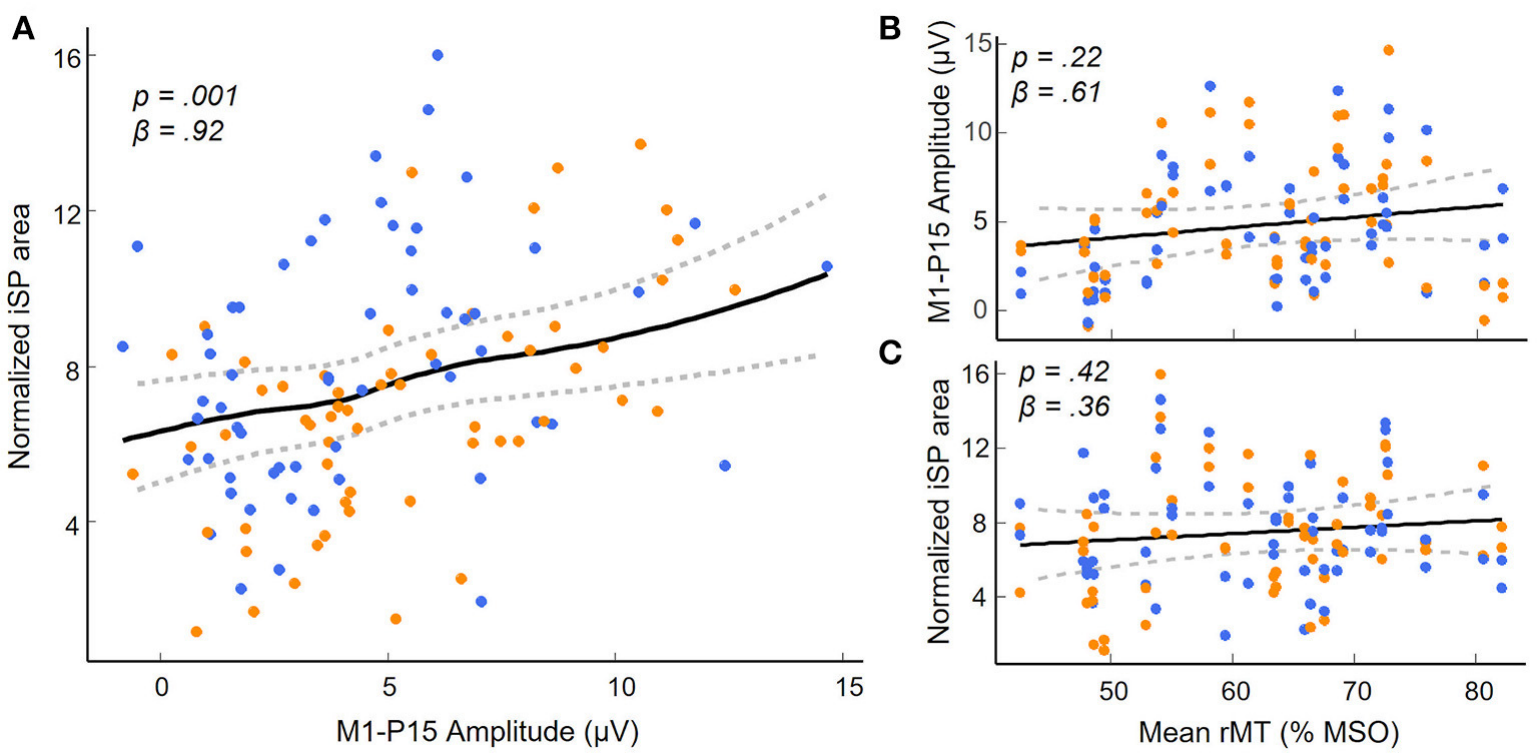
M1-P15 as a measure of transcallosal inhibition. **(A)** Significant positive relationship between M1-P15 amplitude and normalized iSP area, indicating that the larger the M1-P15 amplitude, the greater the iSP. **(B,C)** Results of the control analyses showing non significant relationships between the mean rMT of both hemispheres and the M1-P15 amplitude **(B)** and the normalized iSP area **(C)**. Blue dots indicate LTMS, orange dots indicate RTMS. Fitted curves were drawn by applying a smoothed spline to predicted values in the LMMs obtained by a bootstrap procedure (random sampling with replacement of the subjects; *n* = 500 simulations) using the ‘bootMer' function of the ‘lme4' package in R; dashed lines represent the 95% confidence interval.

**Figure 4:**
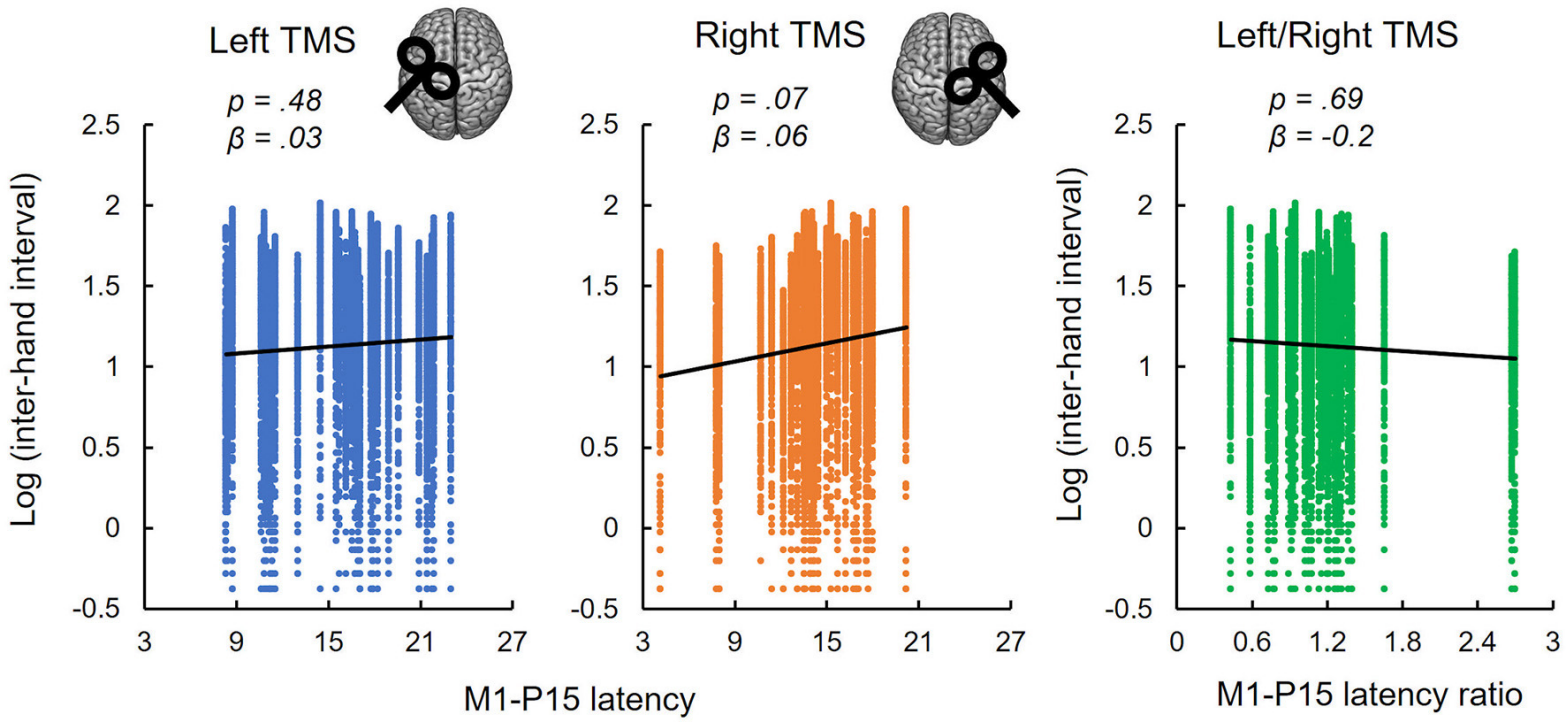
Non-replication of the relationships between M1-P15 latency and bimanual performance. M1-P15 latency recorded during the iSP paradigm was not predictive of bimanual performance in the *Sequence* task, neither after LTMS (left panel), nor after RTMS (central panel) nor the ratio between LTMS and RTMS, as revealed by LMM considering single-trial inter-hand intervals. Fitted lines represent linear trends.

**Figure 5:**
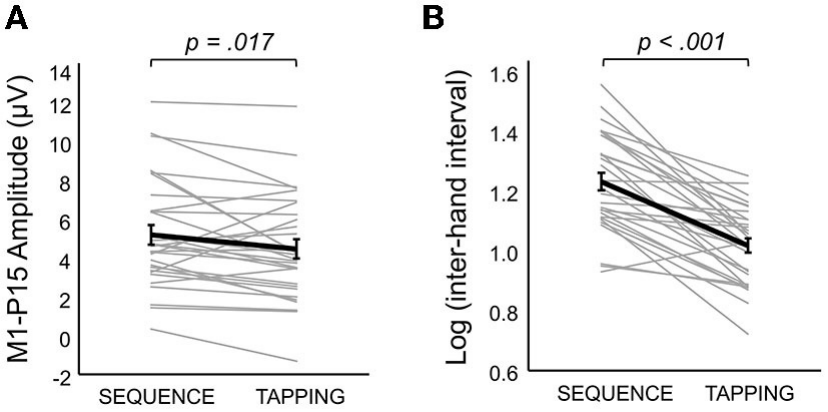
Bimanual task manipulation on M1-P15 amplitude and coordination performance. **(A)** Bimanual task affected M1-P15 amplitude, with a larger M1-P15 recorded during the *Sequence* compared to the *Tapping* task (*p* value is referred to the significant main effect of Task in the 2 × 2 rm-ANOVA with factors Hemisphere and Task). Gray thin lines represent individual data of the mean between LTMS and RTMS; thick black line represents the mean across participants (SE in error bars). **(B)** The inter-hand interval measured during the behavioral *Sequence* task was significantly longer than the one recorded during the behavioral *Tapping* task (*p* value resulting from dependent-samples *t*-test). Gray thin lines represent individual data; thick black line represents the mean across participants (SE in error bars).

(i) As predicted, LMMs showed that M1-P15 amplitude positively predicted the normalized area of the iSP (*t*_(28)_ = 3.3, *p* = 0.001), so that the larger the M1-P15, the stronger the interhemispheric inhibition in the ipsilateral APB (Figure 3A). This relationship was unlikely explained by a third common factor, namely the rMT, as suggested by a non-significant relationship between rMT and P15 amplitude (*t*_(29)_ = 1.26, *p* = 0.22; Figure 3B) and between rMT and iSP normalized area (*t*_(28)_ = 0.83, *p* = 0.42; Figure 3C). Results on the relationship between M1-P15 amplitude and iSP normalized area when calculating the iSP with a minimum duration of 5 ms were similar to the original ones with a minimum duration of 10 ms (*t*_(28)_ = 2.85, *p* = 0.006).
(ii) No evidence was found that bimanual performance in *Sequence* condition was predicted by the latency of M1-P15 for LTMS (*t* = 0.74, *p* = 0.47), RTMS (*t* = 1.9, *p* = 0.07), nor the ratio between the two (*t* = −0.42, *p* = 0.69; Figure 4). Null effects were observed also when considering the M1-P15 latency measured while participants were performing the *Sequence* task (LTMS: *t* = 0.09, *p* = 0.93; RTMS: *t* = 0.74, *p* = 0.48; rate: *t* = −0.76, *p* = 0.46). From the control analysis on the behavioral measure of performance, we excluded an effect of fatigue or learning throughout the behavioral task, because one-way rm-ANOVA with the 3-level factor Block on the inter-hand interval showed a non-significant effect (*F*_(2,58)_ = 0.75, *p* = 0.48). Overall, the M1-P15 latency was unaffected by the factors we manipulated, i.e., Hemisphere, both during bimanual paradigm (*F*_(1,29)_ = 0.02, *p* = 0.9) and during iSP paradigm (*F*_(1,28)_ = 2.13, *p* = 0.16), Task during bimanual paradigm (*F*_(1,29)_ = 0.56, *p* = 0.46) and Contraction during iSP paradigm (*F*_(1,28)_ = 0.47, *p* = 0.5); no interaction were observed (Hemisphere X Task: *F*_(1,29)_ = 1.91, *p* = 0.18; Hemisphere X Contraction: *F*_(1,28)_ = 0.01, *p* = 0.92).
(iii) The iSP paradigm showed no modulation of interhemispheric inhibition by hand contraction for both iSP and M1-P15. The rm-MANOVA revealed no significant main effect of Contraction (MATS_(2)_ = 0.64, *p* = 0.22) nor Contraction X Hemisphere (MATS_(2)_ = 1.00, *p* = 0.53) on the iSP normalized area and M1-P15 amplitude during the iSP paradigm. We observed a main effect of Hemisphere (MATS_(2)_ = 6.66, *p* = 0.023): *Post-hoc* analyses by means of rm-ANOVAs revealed a significant main effect of Hemisphere on iSP only (*F*_(1,28)_ = 4.24, *p* = 0.049), with a larger iSP after RTMS compared to LTMS; this effect did not reach the level of significance on M1-P15 amplitude (*F*_(1,28)_ = 3.69, *p* = 0.065). The control analyses confirmed a non-significant effect of Contraction on the (non-normalized) iSP area (*F*_(1,28)_ = 0.21, *p* = 0.65) and indicated that left and right rMT did not differ (*t*_(29)_ = 0.11, *p* = 0.91), suggesting that the effect of Hemisphere cannot be explained by a difference in TMS intensity. Importantly, the M1-P15 amplitude in the bimanual tasks was modulated according to the movements performed (Significant main effect of Task, *F*_(1,29)_ = 6.43, *p* = 0.017), with a larger M1-P15 recorded during *Sequence* (5.17 ± 0.5 μV) compared to *Tapping* (4.44 ± 0.5 μV; Figure 5A). No significant main effect of Hemisphere (*F*_(1,29)_ = 0.03, *p* = 0.87) nor interaction Task X Hemisphere (*F*_(1,29)_ = 1.79, *p* = 0.19) were observed. Finally, the inter-hand interval was significantly higher (i.e., lower bimanual coordination) in the *Sequence* (25.7 ± 8.6 ms) compared to the *Tapping* task (15.5 ± 5.1 ms; *t*_(29)_ = 8.07, *p* < 0.001; Figure 5B).

Regarding the questionnaire on the sensations associated with TMS, on average the hearing sensation was rated 1.6 ± 0.16 on a Likert scale from 0 (“no noise”) to 4 (“very loud noise”), suggesting that the use of noise-canceling earphones playing white noise did not mask the TMS click completely.

## Discussion

In our previous work (Bortoletto et al., 2021), we suggested that the amplitude of M1-P15 represents the inhibition conveyed by the stimulated M1 to its homologue along the fibers of the corpus callosum, by reporting a significant relationship between the amplitude of M1-P15 and of the iSP—a well-known peripheral measure of interhemispheric inhibition (Ferbert et al., 1992; Kuo et al., 2017). In the present study, we provide further evidence for validating the M1-P15 as index of transcallosal inhibition and we show its modulation during task execution. By corroborating our knowledge on the physiological meaning of the M1-P15, we set the bases for the development of a physiological biomarker that can be applied in neuropsychiatric disorders including alterations in effective connectivity, such as demyelinating diseases and dementia (Wahl et al., 2016; Bagattini et al., 2019).

First, we show a replication of the relationship between M1-P15 and iSP, such that the larger the M1-P15, the greater the normalized iSP area. Importantly, control analyses ruled out the risk of an artifactual or sensory contamination that could have explained the relationship between the two measures, showing that neither M1-P15 amplitude nor iSP normalized area were associated with TMS intensity (Niessen et al., 2021). The characterization of early TEP components as reflecting contralateral activation in the motor network is consistent with previous TMS-EEG studies, in which brain source modeling localized the response in the first tens of ms after left M1 stimulation in the right M1 (Ilmoniemi et al., 1997; Zazio et al., 2021), and more generally with findings from double-coil paradigms (Ferbert et al., 1992; Ni et al., 2020). Nevertheless, the present results take this body of evidence one step forward, characterizing a specific feature of a TEP component, namely the M1-P15 amplitude, as a reliable cortical measure of the strength of transcallosal inhibition of contralateral motor areas. Indeed, the hypothesis that the iSP is conveyed by transcallosal cortical fibers rather than by uncrossed ipsilateral cortico-spinal pathways has been supported since very early studies, based on the latency iSP (Wassermann et al., 1991; Ferbert et al., 1992), especially for the APB muscle (Jung and Ziemann, 2006), and on data from patients with abnormalities of the corpus callosum (Meyer et al., 1995). Interestingly, it has been recently suggested that the reduced EMG activity observed in the ipsilateral muscles after M1-TMS may not reflect a general and undifferentiated inhibition between motor cortices, but it may arise from a more complex integrative function ensured by mechanisms of surrounding inhibition (Carson, 2020). Therefore, the same reasoning may apply to M1-P15: although it is associated with an inhibitory effect recorded on ipsilateral muscles, it cannot be excluded that M1-P15 may also subtend narrowed excitatory effects. Finally, it is important to stress that the link between M1-P15 and the iSP, although reliable, is correlative in nature, and thus further research is needed to show causal evidence.

Then, we investigated whether we could reproduce the predictive value of M1-P15 latency recorded during the iSP paradigm on bimanual coordination in sequential thumb-to-finger opposition movements. Contrary to our expectations, we did not observe any significant relationship between the M1-P15 latency and behavioral performance at the *Sequence* task. Control analyses on behavioral performance throughout the experimental blocks rule out possible confounding effects of learning on fatigue that may have impacted the performance when considering all blocks together. A possible reason that may explain this null result is that the conditions in which M1-P15 was recorded during the iSP paradigm were not identical to our previous study (Bortoletto et al., 2021). Beside the classical iSP condition in which the contralateral hand was at rest (Kuo et al., 2017), in both studies we recorded a condition in which the contralateral hand was motorically active, expecting to increase the interhemispheric inhibition (Giovannelli et al., 2009): in Bortoletto et al. (2021) participants were involved in visually-cued thumb-to-finger opposition (*Task*); In the present study, they were asked to maintain a certain level of muscle contraction (*Contracted*). Moreover, the relationship between M1-P15 and bimanual coordination was investigated for the M1-P15 recorded during the same bimanual movements performed in the behavioral tasks. Nevertheless, even in this case we did not observe a significant relationship between M1-P15 latency and behavioral performance. It has to be noted that in Bortoletto et al. (2021) the TMS was delivered at the time of movement initiation, which may be a critical timing for M1-M1 interaction, while here it was delivered randomly between one finger movement and the following one. Overall, the discrepancies between this study and our previous one highlight the need for future investigations on the relationship between TCD and bimanual coordination.

The choice of delivering TMS pulses at random time intervals between the finger movements was important to avoid auditory-evoked potentials associated with the metronome but it may have introduced variability in the recoding of M1-P15, thus hindering possible relationships with the behavioral performance. To this regard, although the results from the pilot study showed that on average the M1-P15 was detectable in every time interval, the small sample size prevented to statistically test for possible modulations of M1-P15 depending on the time interval, which is an intriguing aspect that may be tested in future studies. Furthermore, stimulating at 110% while performing motor tasks was expected to induce MEPs. However, this issue was not expected to interfere with the aim of the study, for two reasons. First, the performance at the bimanual tasks was not analyzed during the TMS-EEG recording (i.e., when it could be impaired by MEPs), but during the behavioral tasks, instead. Second, the reafference of the motor twitches on TEPs is expected to occur approximately at 60 ms after the TMS pulse (Petrichella et al., 2017), thus not interfering with the early M1-P15.

Finally, the aim of modulating M1-P15 according to the state of the motor cortex was fulfilled in an exploratory way.

In both the TMS-EEG recordings (i.e., during bimanual tasks and during the iSP paradigm), the within-subject design including a task modulation represents a powerful strategy to face the problem of confounds deriving from sensory contamination in TMS-evoked potentials. Indeed, the experimental conditions only differ on the task the participants were performing, but they are identical in the stimulation parameters (i.e., stimulation site and TMS intensity), therefore excluding that possible differences in TEPs between conditions may be due to differences in TMS-induced artifacts or sensory contaminations.

The iSP paradigm was found to be inadequate for the aim, as the iSP was not modulated by hand contraction. In fact, during the iSP paradigm we manipulated the activity of the contralateral hand, expecting to induce a stronger interhemispheric inhibition in the *Contracted* compared to the *Relaxed* condition (Giovannelli et al., 2009), which we did not observe. The null result on iSP modulation was surprising, considering the consistent effects reported previously throughout several manipulations of contralateral hand activity (Giovannelli et al., 2009). However, even in our previous study (Bortoletto et al., 2021) we did not observe a modulation of iSP depending on contralateral hand activity (no main effect of Task, *p* = 0.86). The negative finding of the present work cannot depend on the quantification method for the iSP, i.e., the normalized iSP area *vs*. the iSP area used in Giovannelli et al. (2009), as it was confirmed by a control analysis on the iSP area. One possible confound cannot be fully ruled out is the induction of fatigue in our iSP paradigm, although we employed multiple strategies to avoid it (i.e., breaks were provided in the middle of the blocks and participants were instructed to relax their hands whenever they felt fatigue). Therefore, further research is needed to understand the nature of this inconsistency. Nevertheless, considering that the magnitude of the iSP and the amplitude of the M1-P15 are expected to reflect the same process, i.e., the strength of interhemispheric inhibition, it is not surprising that even M1-P15 amplitude was unaffected by contralateral hand activity.

Importantly, looking at the bimanual tasks, we found a modulation of the behavioral performance and of the M1-P15 amplitude, with a larger M1-P15 amplitude during the sequential (*Sequence*) compared to the repetitive (*Tapping*) bimanual movements. This result represents the first evidence of a task-dependent modulation of M1-P15, further suggesting the physiological, non-artifactual nature of this early TEP component. The presence of the M1-P15 during the bimanual tasks, with topographical patterns comparable to the ones observed during the iSP paradigm, suggests that the transcallosal information transfer conveys an inhibitory function also during the execution of bimanual movements, supporting the hypothesis that functional inhibition of contralateral M1 may be needed to ensure neural cross-talk at the cortico-spinal level (but see Carson, 2020). Furthermore, the modulation of M1-P15 with movement complexity suggests that the execution of sequential bimanual movements requires stronger interhemispheric inhibition compared to repetitive bimanual movements. Intriguingly, this hypothesis is in line with evidence on patients with multiple sclerosis (Bonzano et al., 2008) and with callosotomy and agenesis of the corpus callosum (Eliassen et al., 2000; Chen et al., 2005), showing that sequential but not repetitive finger opposition movements rely on callosal integrity. Therefore, M1-P15 may represent a mechanism of transcallosal inhibition that is modulated during execution of complex movements but not during force control.

## Conclusions

Taken together, our findings support the M1-P15, an early TEP component after M1 stimulation, as a cortical index of interhemispheric inhibition between motor cortices, as revealed by the successful replication of its positive relationship with the iSP. Furthermore, we introduced novel evidence of a task-dependent modulation of M1-P15 amplitude during bimanual tasks, likely depending on the involvement of transcallosal connections for task execution. In future studies, further investigation is required to understand the relationship between TCD and bimanual coordination, as well as the effects of contralateral hand activity on interhemispheric inhibition during iSP paradigms.

To the best of our knowledge the present study is the first example of preregistration in the TMS-EEG field. While certainly challenging especially for the technical aspects of TMS-EEG coregistration, preregistration of TMS-EEG studies is feasible and thus should be considered in future studies as a powerful strategy to increase the methodological rigor and transparency and to provide an unbiased picture of the results, eventually leading to an improvement of research quality.

## Data availability statement

The data supporting the conclusions of this article will be openly available at: https://gin.g-node.org/AgneseZazio/Zazio-FHN2022.

## Ethics statement

The studies involving human participants were reviewed and approved by Ethical Committee of the IRCCS Istituto Centro San Giovanni di Dio Fatebenefratelli (Brescia, 54-2019). The patients/participants provided their written informed consent to participate in this study.

## Author contributions

AZ, GB, and MB conceived and designed the study. GB wrote the scripts for the experimental paradigms and built custom devices. AZ, GB, and EM collected the data. AZ and GB performed the signal processing. AZ and CF planned and performed the statistical analyses. AZ took care of data visualization. AZ and MB managed data curation, supervised the research activity, and wrote the first draft of the manuscript. All authors contributed to manuscript revision, read, and approved the submitted version.

## Funding

This study was supported by the Italian Ministry of Health funding Ricerca Corrente and by the Department of Philosophy Piero Martinetti of the University of Milan with the Project Departments of Excellence 2018–2022 awarded by the Italian Ministry of Education, University and Research (MIUR) to GB.

## Conflict of interest

The authors declare that the research was conducted in the absence of any commercial or financial relationships that could be construed as a potential conflict of interest.

## Publisher's note

All claims expressed in this article are solely those of the authors and do not necessarily represent those of their affiliated organizations, or those of the publisher, the editors and the reviewers. Any product that may be evaluated in this article, or claim that may be made by its manufacturer, is not guaranteed or endorsed by the publisher.

## References

[B1] Awiszus F. (2003). TMS and threshold hunting. Suppl Clin Neurophysiol. 56:13–23. 10.1016/s1567-424x(09)70205-3 14677378

[B2] Awiszus F. (2011). Fast estimation of transcranial magnetic stimulation motor threshold: is it safe? Brain Stimul. 4, 58–59. 10.1016/j.brs.2010.09.004 21255757

[B3] Bagattini C. Mutanen T. P. Fracassi C. Manenti R. Cotelli M. Ilmoniemi R. J.. (2019). Predicting Alzheimer's disease severity by means of TMS–EEG coregistration. Neurobiol. Aging 80, 38–45. 10.1016/j.neurobiolaging.2019.04.008 31077959

[B4] Bonato C. Miniussi C. Rossini P. M. (2006). Transcranial magnetic stimulation and cortical evoked potentials: a TMS/EEG co-registration study. Clin. Neurophysiol. 117, 1699–1707. 10.1016/j.clinph.2006.05.006 16797232

[B5] Bonzano L. Tacchino A. Roccatagliata L. Abbruzzese G. Mancardi G. L. Bove M. (2008). Callosal contributions to simultaneous bimanual finger movements. J. Neurosci. 28, 3227–3233. 10.1523/JNEUROSCI.4076-07.2008 18354026 PMC6670695

[B6] Bortoletto M. Bonzano L. Zazio A. Ferrari C. Pedullà L. Gasparotti R.. (2021). Asymmetric transcallosal conduction delay leads to finer bimanual coordination. Brain Stimul. 14, 379–388. 10.1016/j.brs.2021.02.002 33578035

[B7] Bortoletto M. Veniero D. Thut G. Miniussi C. (2015). The contribution of TMS—EEG coregistration in the exploration of the human cortical connectome. Neurosci. Biobehav. Rev. 49, 114–124. 10.1016/j.neubiorev.2014.12.014 25541459

[B8] Button K. S. Ioannidis J. P. A. Mokrysz C. Nosek B. A. Flint J. Robinson E. S. J.. (2013). Power failure: why small sample size undermines the reliability of neuroscience. Nat. Rev. Neurosci. 14, 365–376. 10.1038/nrn3475 23571845

[B9] Caminiti R. Carducci F. Piervincenzi C. Battaglia-Mayer A. Confalone G. Visco-Comandini F.. (2013). Diameter, length, speed, and conduction delay of callosal axons in macaque monkeys and humans: comparing data from histology and magnetic resonance imaging diffusion tractography. J. Neurosci. 33, 14501–14511. 10.1523/JNEUROSCI.0761-13.2013 24005301 PMC6618375

[B10] Carson R. G. (2020). Inter-hemispheric inhibition sculpts the output of neural circuits by co-opting the two cerebral hemispheres. J. Physiol. 598, 4781–4802. 10.1113/JP279793 32770748

[B11] Casula E. P. Maiella M. Pellicciari M. C. Porrazzini F. D'Acunto A. Rocchi L.. (2020). Novel TMS-EEG indexes to investigate interhemispheric dynamics in humans. Clin. Neurophysiol. 131, 70–77. 10.1016/j.clinph.2019.09.013 31756594

[B12] Casula E. P. Pellicciari M. C. Bonnì S. Spanò B. Ponzo V. Salsano I.. (2021). Evidence for interhemispheric imbalance in stroke patients as revealed by combining transcranial magnetic stimulation and electroencephalography. Hum. Brain Mapp. 42, 1343–1358. 10.1002/hbm.25297 33439537 PMC7927297

[B13] Chen J. T. Lin Y. Y. Shan D. E. Wu Z. A. Hallett M. Liao K. K. (2005). Effect of transcranial magnetic stimulation on bimanual movements. J. Neurophysiol. 93, 53–63. 10.1152/jn.01063.2003 15331622

[B14] Delorme A. Makeig S. (2004). EEGLAB: an open source toolbox for analysis of single-trial EEG dynamics including independent component analysis. J. Neurosci. Methods 134, 9–21. 10.1016/j.jneumeth.2003.10.009 15102499

[B15] Eliassen J. C. Baynes K. Gazzaniga M. S. (2000). Anterior and posterior callosal contributions to simultaneous bimanual movements of the hands and fingers. Brain 123, 2501–2511. 10.1093/brain/123.12.2501 11099451

[B16] Esposito R. Bortoletto M. Zacà D. Avesani P. Miniussi C. (2022). An integrated TMS-EEG and MRI approach to explore the interregional connectivity of the default mode network. Brain Struct. Funct. 227, 1133–1144. 10.1007/s00429-022-02453-6 35119502 PMC8930884

[B17] Ferbert A. Priori A. Rothwell J. C. Day B. L. Colebatch J. G. Marsden C. D. (1992). Interhemispheric inhibition of the human motor cortex. J. Physiol. 453, 525–546. 10.1113/jphysiol.1992.sp019243 1464843 PMC1175572

[B18] Friston K. Moran R. Seth A. K. (2013). Analysing connectivity with Granger causality and dynamic causal modelling. Curr. Opin. Neurobiol. 23, 172–178. 10.1016/j.conb.2012.11.010 23265964 PMC3925802

[B19] Giovannelli F. Borgheresi A. Balestrieri F. Zaccara G. Viggiano M. P. Cincotta M.. (2009). Modulation of interhemispheric inhibition by volitional motor activity: an ipsilateral silent period study. J. Physiol. 587, 5393–5410. 10.1113/jphysiol.2009.175885 19770195 PMC2793872

[B20] Hui J. Zomorrodi R. Lioumis P. Salavati B. Rajji T. K. Chen R.. (2020). Pharmacological mechanisms of interhemispheric signal propagation: a TMS-EEG study. Neuropsychopharmacology 45, 932–939. 10.1038/s41386-019-0468-7 31357206 PMC7162860

[B21] Ilmoniemi R. J. Hernandez-Pavon J. C. Mäkelä N. N. Metsomaa J. Mutanen T. P. Stenroos M.. (2015). Dealing with artifacts in TMS-evoked EEG. In 37th Annual International Conference of the IEEE Engineering in Medicine and Biology Society (EMBC). IEEE, 230–233. 10.1109/EMBC.2015.7318342 26736242

[B22] Ilmoniemi R. J. Virtanen C. A. J. Ruohonen J. Karhu J. Aronen H. J. Näätänen R.. (1997). Neuronal responses to magnetic stimulation reveal cortical reactivity and connectivity. Neuroreport 8, 3537–3540. 10.1097/00001756-199711100-00024 9427322

[B23] Jarczok T. A. Fritsch M. Kröger A. Schneider A. L. Althen H. Siniatchkin M.. (2016). Maturation of interhemispheric signal propagation in autism spectrum disorder and typically developing controls: a TMS-EEG study. J. Neural Transm. 123, 925–935. 10.1007/s00702-016-1550-5 27177879

[B24] Jung P. Ziemann U. (2006). Differences of the ipsilateral silent period in small hand muscles. Muscle Nerve 34, 431–436. 10.1002/mus.20604 16810689

[B25] Koganemaru S. Mima T. Nakatsuka M. Ueki Y. Fukuyama H. Domen K. (2009). Human motor associative plasticity induced by paired bihemispheric stimulation. J. Physiol. 587, 4629–4644. 10.1113/jphysiol.2009.174342 19687124 PMC2768018

[B26] Kuo Y. L. Dubuc T. Boufadel D. F. Fisher B. E. (2017). Measuring ipsilateral silent period: effects of muscle contraction levels and quantification methods. Brain Res. 1674, 77–83. 10.1016/j.brainres.2017.08.015 28823955

[B27] Massimini M. Ferrarelli F. Huber R. Esser S. K. Singh H. Tononi G. (2005). Breakdown of cortical effective connectivity during sleep. Science 309, 2228–2232. 10.1126/science.1117256 16195466

[B28] Meyer B. U. Röricht S. Von Einsiedel H. G. Kruggel F. Weindl A. (1995). Inhibitory and excitatory interhemispheric transfers between motor cortical areas in normal humans and patients with abnormalities of the corpus callosum. Brain 118, 429–440. 10.1093/brain/118.2.429 7735884

[B29] Miniussi C. Thut G. (2010). Combining TMS and EEG offers new prospects in cognitive neuroscience. Brain Topogr. 22, 249–256. 10.1007/s10548-009-0083-8 19241152

[B30] Momi D. Ozdemir R. A. Tadayon E. Boucher P. Di Domenico A. Fasolo M.. (2021a). Perturbation of resting-state network nodes preferentially propagates to structurally rather than functionally connected regions. Sci. Rep. 11, 1–11. 10.1038/s41598-021-90663-z 34127688 PMC8203778

[B31] Momi D. Ozdemir R. A. Tadayon E. Boucher P. Shafi M. M. Pascual-Leone A.. (2021b). Network-level macroscale structural connectivity predicts propagation of transcranial magnetic stimulation. Neuroimage 229, 117698. 10.1016/j.neuroimage.2020.117698 33385561 PMC9094638

[B32] Morishima Y. Akaishi R. Yamada Y. Okuda J. Toma K. Sakai K. (2009). Task-specific signal transmission from prefrontal cortex in visual selective attention. Nat. Neurosci. 12, 85–91. 10.1038/nn.2237 19098905

[B33] Mutanen T. P. Kukkonen M. Nieminen J. O. Stenroos M. Sarvas J. Ilmoniemi R. J. (2016). Recovering TMS-evoked EEG responses masked by muscle artifacts. Neuroimage 139, 157–166. 10.1016/j.neuroimage.2016.05.028 27291496

[B34] Mutanen T. P. Metsomaa J. Liljander S. Ilmoniemi R. J. (2018). Automatic and robust noise suppression in EEG and MEG: the SOUND algorithm. Neuroimage 166, 135–151. 10.1016/j.neuroimage.2017.10.021 29061529

[B35] Ni Z. Leodori G. Vial F. Zhang Y. Avram A. V. Pajevic S.. (2020). Measuring latency distribution of transcallosal fibers using transcranial magnetic stimulation. Brain Stimul. 13, 1453–1460. 10.1016/j.brs.2020.08.004 32791313 PMC7417270

[B36] Niessen E. Bracco M. Mutanen T. P. Robertson E. M. (2021). An analytical approach to identify indirect multisensory cortical activations elicited by TMS? Brain Stimul. 14, 276–378. 10.1016/j.brs.2021.02.003 33581281

[B37] Oostenveld R. Fries P. Maris E. Schoffelen J. M. (2011). FieldTrip: open source software for advanced analysis of MEG, EEG, and invasive electrophysiological data. Comput. Intell. Neurosci. 2011:156869. 10.1155/2011/156869 21253357 PMC3021840

[B38] Paul M. Govaart G. H. Schettino A. (2021). Making ERP research more transparent: guidelines for preregistration. Int. J. Psychophysiol. 164, 52–63. 10.1016/j.ijpsycho.2021.02.016 33676957

[B39] Pavlov Y. G. Adamian N. Appelhoff S. Arvaneh M. Benwell C. S. Y. Beste C.. (2021). #EEGManyLabs: Investigating the replicability of influential EEG experiments. Cortex 144, 213–229. 10.1016/j.cortex.2021.03.013 33965167

[B40] Pentland A. (1980). Maximum likelihood estimation: the best PEST. Percept. Psychophys. 28, 377–379. 10.3758/BF03204398 7465322

[B41] Petrichella S. Johnson N. He B. (2017). The influence of corticospinal activity on TMS-evoked activity and connectivity in healthy subjects: a TMS-EEG study. PLoS ONE 12, 1–18. 10.1371/journal.pone.0174879 28384197 PMC5383066

[B42] Poldrack R. A. Baker C. I. Durnez J. Gorgolewski K. J. Matthews P. M. Munafò M. R.. (2017). Scanning the horizon: towards transparent and reproducible neuroimaging research. Nat. Rev. Neurosci. 18, 115–126. 10.1038/nrn.2016.167 28053326 PMC6910649

[B43] R Core Team. (2020). R: A Language and Environment for Statistical Computing (Version 4.0). Available online at: https://www.R-project.org/

[B44] Ringo J. L. Doty R. W. Demeter S. Simard P. Y. (1994). Time is of the essence: a conjecture that hemispheric specialization arises from interhemispheric conduction delay. Cereb. Cortex 4, 331–343. 10.1093/cercor/4.4.331 7950307

[B45] Rossi S. Antal A. Bestmann S. Bikson M. Brewer C. Brockmöller J.. (2020). Safety and recommendations for TMS use in healthy subjects and patient populations, with updates on training, ethical and regulatory issues: expert guidelines. Clin. Neurophysiol. 132, 269–306. 10.1016/j.clinph.2020.10.003 33243615 PMC9094636

[B46] The jamovi project. (2021). jamovi (Version 1.6). Available online at: https://www.jamovi.org

[B47] Trompetto C. Bove M. Marinelli L. Avanzino L. Buccolieri A. Abbruzzese G. (2004). Suppression of the transcallosal motor output: a transcranial magnetic stimulation study in healthy subjects. Exp. Brain Res. 158, 133–140. 10.1007/s00221-004-1881-6 15118793

[B48] Veniero D. Bortoletto M. Miniussi C. (2009). TMS-EEG co-registration : on TMS-induced artifact. Clin. Neurophysiol. 120, 1392–1399. 10.1016/j.clinph.2009.04.023 19535291

[B49] Voineskos A. N. Farzan F. Barr M. S. Lobaugh N. J. Mulsant B. H. Chen R.. (2010). The role of the corpus callosum in transcranial magnetic stimulation induced interhemispheric signal propagation. Biol. Psychiatry 68, 825–831. 10.1016/j.biopsych.2010.06.021 20708172

[B50] Wahl M. Lauterbach-Soon B. Hattingen E. Hübers A. Ziemann U. (2016). Callosal anatomical and effective connectivity between primary motor cortices predicts visually cued bimanual temporal coordination performance. Brain Struct. Funct. 221, 3427–3443. 10.1007/s00429-015-1110-z 26365504

[B51] Wassermann E. M. Fuhr P. Cohen L. G. Hallet M. (1991). Effects of transcranial magnetic stimulation on ipsilateral muscles. Neurology 41, 17959–11799. 10.1212/WNL.41.11.1795 1944911

[B52] Zazio A. Miniussi C. Bortoletto M. (2021). Alpha-band cortico-cortical phase synchronization is associated with effective connectivity in the motor network. Clin. Neurophysiol. 132, 2473–2480. 10.1016/j.clinph.2021.06.025 34454275

[B53] Zwaan R. A. Etz A. Lucas R. E. Donnellan M. B. (2018). Making replication mainstream. Behav. Brain Sci. 41, 1–50. 10.1017/S0140525X17001972 29065933

