# Supplementary material for: M1-P15 as a cortical marker for transcallosal inhibition: A preregistered TMS-EEG study
Source: Front Hum Neurosci. 2022 Sep 16;16:937515. doi: 10.3389/fnhum.2022.937515 (PMC9523880; doi:10.3389/fnhum.2022.937515)
Supplement: Supplementary file 1 [file Data_Sheet_1.pdf]

Supplementary Materials for

M1-P15 as a cortical marker for transcallosal inhibition: a preregistered TMS-EEG study

Agnese Zazio[1,*], Guido Barchiesi[1,2], Clarissa Ferrari[4], Eleonora Marcantoni[1], Marta Bortoletto[1]

[1] Neurophysiology Lab, IRCCS Istituto Centro San Giovanni di Dio Fatebenefratelli, Brescia (Italy)

[2] Cognition in Action (CIA) Unit - PHILAB, Department of Philosophy, University of Milan, Milan (Italy)

[4] Statistics Unit, IRCCS Istituto Centro San Giovanni di Dio Fatebenefratelli, Brescia (Italy)

*** Corresponding author:**

Dr Agnese Zazio

Neurophysiology Lab, IRCCS Istituto Centro San Giovanni di Dio Fatebenefratelli

Via Pilastroni 4, 25125 Brescia, Italy

Tel.: +390303501596

E-mail address: agnese.zazio@cognitiveneuroscience.it

Pilot experiment

A pilot experiment was performed before data collection with the following aims: P1) ensuring the feasibility of the new finger-sensors to measure behavioral performance in unimanual and bimanual movements, P2) ensuring the presence of M1-P15 during bimanual movements in TMS-EEG recording, and P3) identifying the optimal time range for TMS delivery during bimanual movements. Furthermore, we used the data of two pilot subjects to qualitatively compare the TEPs obtained after high-pass filters at 1 Hz and at 0.1 Hz.

Participants

Six young healthy participants (3 women, mean age ± SE: 28 ± 2.1 years, right handed). The same participants were not enrolled in the main experiment.

Design and procedure

Participants underwent a single-session within-subject design, including behavioral tasks and a TMS-EEG recording during the bimanual *Sequence* task. The behavioral tasks were identical to the one of the main experiment. In the TMS-EEG recording, 20 blocks of single pulse TMS were delivered over the left and the right M1 at 110% of rMT (10 blocks per hemisphere). In each block, TMS was delivered at 30 different time intervals after the metronome sound (from 0 to 500 ms, aligned with refresh rate, steps of 16.7 ms). On average, TMS was delivered every 4 movements; the ISI between TMS pulses was 1 s. Since the randomization of the TMS pulses associated with metronome sounds did not guarantee that the time interval between two consecutive TMS was at least 1 s, we added 2 sounds (i.e., 1 s) between those separated by less than 1s; therefore, the total number of the metronome sounds can slightly vary among blocks and participants. TEPs were analyzed by averaging over 5 time windows (i.e, 0-100 ms, 100-200 ms, 200-300 ms, 300-400 ms, 400-500 ms after the metronome sound), each comprising 6 intervals for TMS delivery, and resulting in a total of 60 TMS pulses for each time window and hemisphere (Figure S1A).

Analysis

TMS-EEG data was analyzed following the same pipeline described for the main experiment. On the data of two subjects, we run a control analysis in which the frequency of the high-pass filter was set at 0.1 Hz.

Results

Results of the pilot experiment were the following: P1) the recording of the first 3 participants revealed a few technical problems with the new finger-sensors, which turned out to be too thick and did not make good contact; therefore, we created new thinner sensors for the remaining participants, for which we successfully measured a clean signal for both bimanual and unimanual touches with a sampling rate of 9600 Hz; P2) M1-P15, defined as a contralateral positive component peaking ~15 ms after the TMS pulse, was present also during bimanual thumb-to-finger sequential movements (Figure S1B); P3) M1-P15 was present across all intervals of TMS delivery (Figure S1C).

In summary, the pilot experiment enabled us to improve our new finger-sensors and to ensure that M1-P15 is present also during bimanual thumb-to-finger opposition movements independently of the timing of TMS pulses after the metronome sound. Therefore, in the TMS-EEG recording during bimanual tasks of the main experiment TMS pulses were randomly delivered in the time interval between the metronome sounds.

Figures (Pilot experiment)

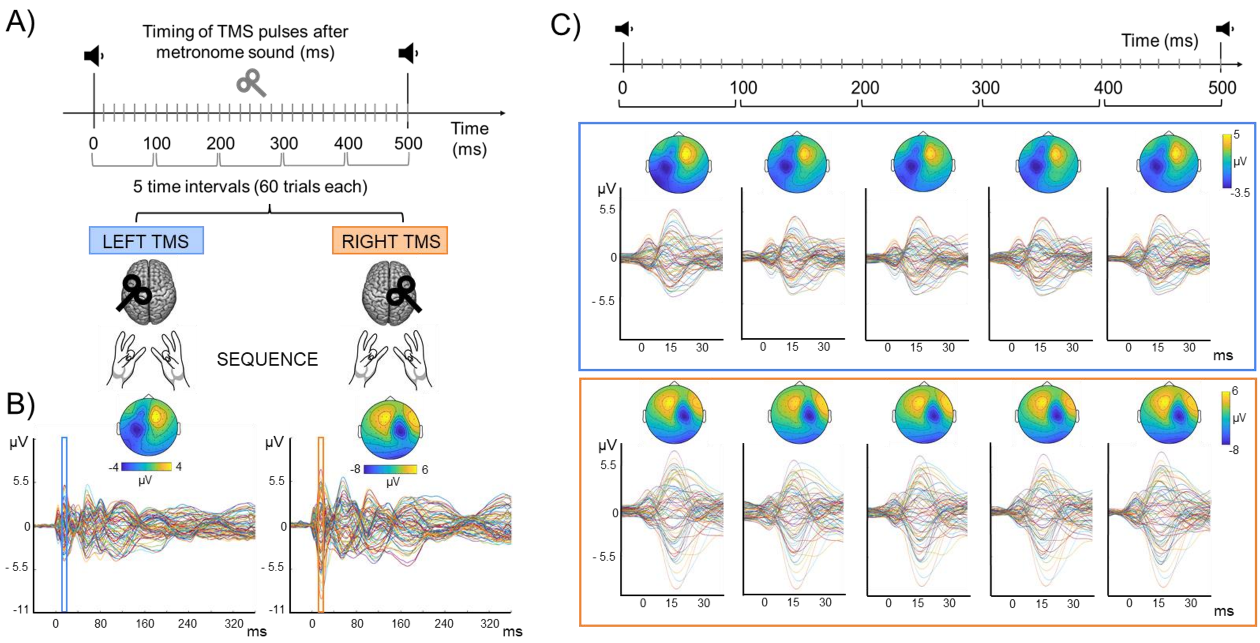

Figure S1. Pilot experiment methods and results. **A)** Schematic representation of the experimental design: while participants were performing the *Sequence* task, TMS was delivered in 30 possible time intervals from the metronome sound, that can be grouped into 5 time intervals of 60 trials each. Both hemispheres were stimulated in different blocks, in counterbalanced order among participants. **B)** Grand average of TEPs and topographical maps after LTMS (left panel) and RTMS (right panel), merging all time intervals. Topographies were obtained by averaging over time between 10 and 20 ms after the TMS pulse; amplitude range is shown in colorbars. **C)** Grand average of TEPs and topographical maps for each of the 5 time intervals of TMS delivery after the metronome sound, for LTMS (upper panel) and RTMS (lower panel), showing no macroscopical modulation of M1-P15 among the different intervals. Amplitude range of topographical maps is shown in colorbars.

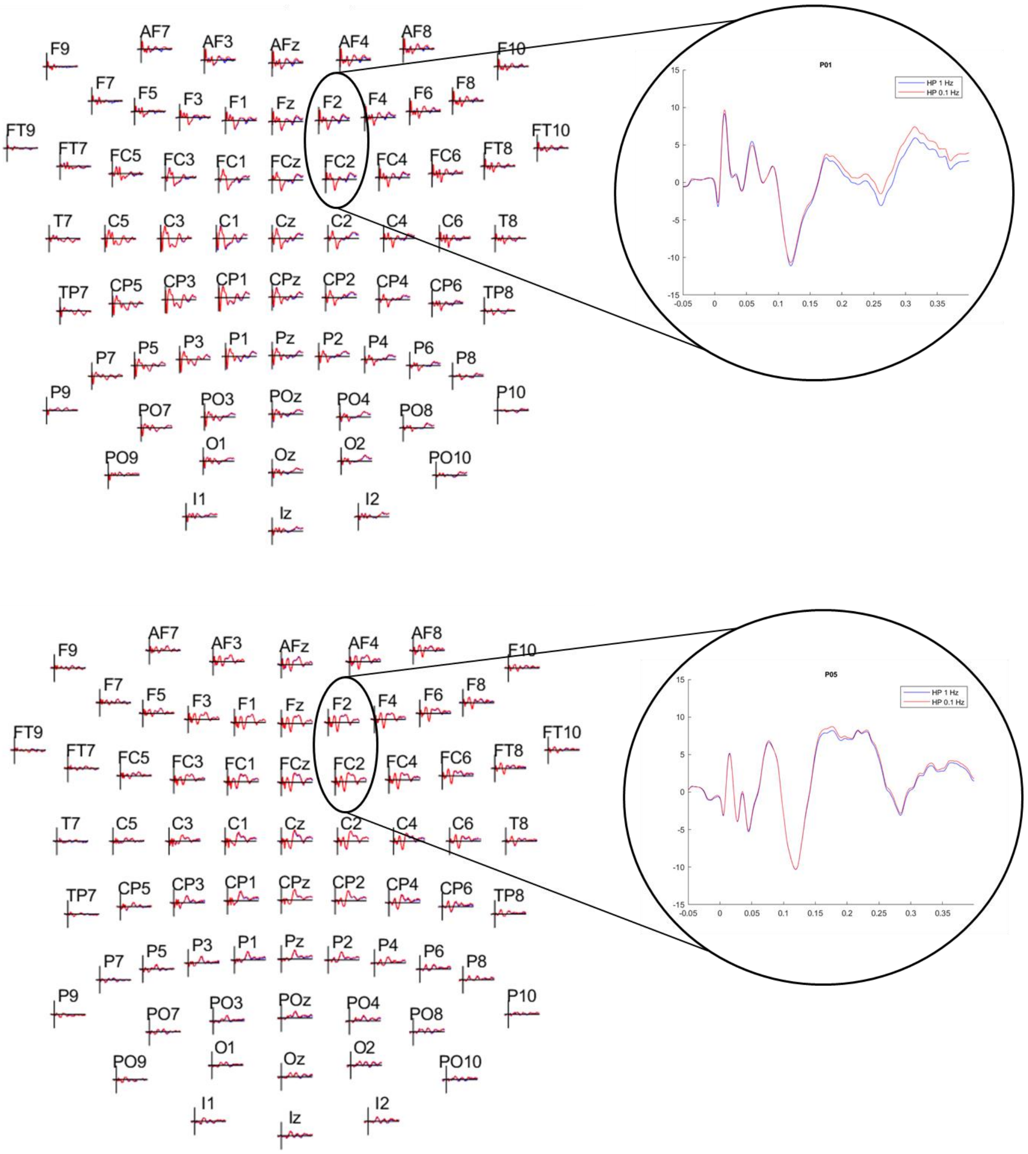

Figure S2. Comparison between high-pass filtering at 1 Hz and 0.1 Hz in two pilot subjects. Average over trials showing the overlay of TEPs obtained after 1 Hz filter (blue trace) and 0.1 Hz filter (red trace). The enlargement on the right shows the average signal between the two electrodes considered for M1-P15 after LTMS (i.e., F2, FC2).

Main experiment

Sample size estimation

A formal power analysis was based on the results already published by the same research group in a comparable study design (Bortoletto *et al.*, 2021). In detail, using the '*powerSim*' function of the R library '*simr*' a post-hoc power analysis was performed on the linear mixed models (LMM) in Table S4 of such paper, obtaining values ranged from β=0.68 (for the LMM assessing the relation between 'Interhand interval (thumb-to-index repetitions)' and M1-P15 amplitude) to β=0.92 (for the LMM assessing the relation between 'iSP normalized area' and M1-P15 amplitude). The minimum sample size for this study was computed in order to reach a sufficient power (set equal to 0.8) for all performed models. Specifically, through the function *powerSim* applied on the LMMs of Bortoletto et al. 2021 and by setting a power β=0.8 and a significance level alpha =0.05; the minimum sample obtained was N=25.

In addition, a further power analysis was performed for the MANOVA model. Using data in Bortoletto et al. (2021) a Pillai V effect size=0.2 was obtained by applying a MANOVA on 'iSP normalized area' and M1-P15 amplitude as dependent variables and 'Condition' (factor at 4 levels) as independent variable (see model *Ia* in Table S4 in Bortoletto *et al.*, 2021). With such a value of Pillai V, the corresponding effect size f = V/(1-V)=0.25 was computed and used as input for the sample size computation carried out by the tool G*Power (for MANOVA: Repeated measures – within factors). Thus, with an effect size f=0.25 (medium effect size), a power β=0.8, a significance level alpha=0.05 and hypothesizing a repeated measure correlation equal to 0.5, the minimum estimated sample size was N=28.

The sample size N=32 of the current study, allows to reach adequate power for all the models performed.

Figures (main experiment)

Fig. S3

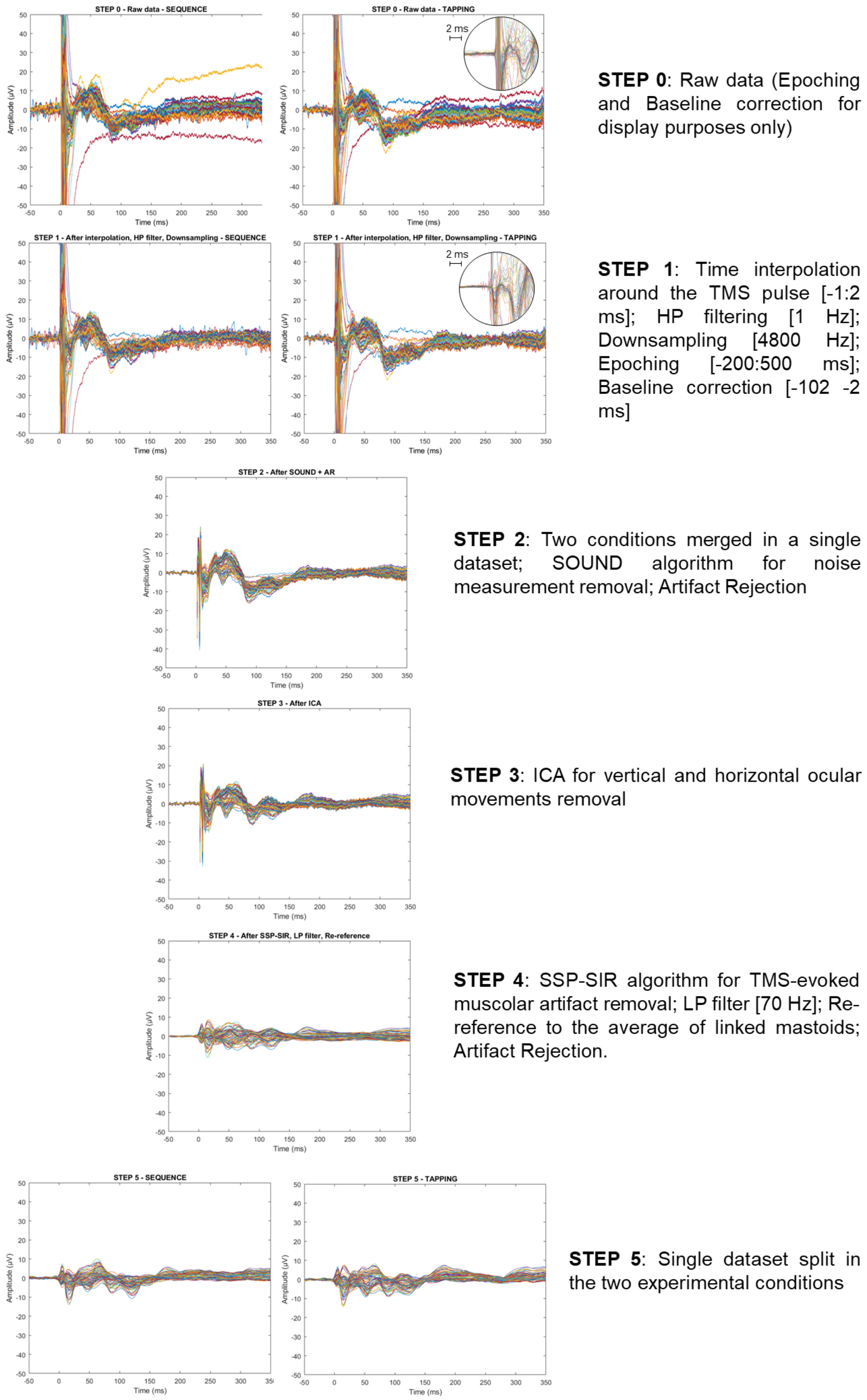

Fig. S3 Effect of the main steps of preprocessing on TMS-EEG data of a single participant recorded during the bimanual tasks after LTMS. Single butterfly plots show the average over trials of all EEG channels after the preprocessing steps outlined on the right side of the figure.

Fig. S4

A)

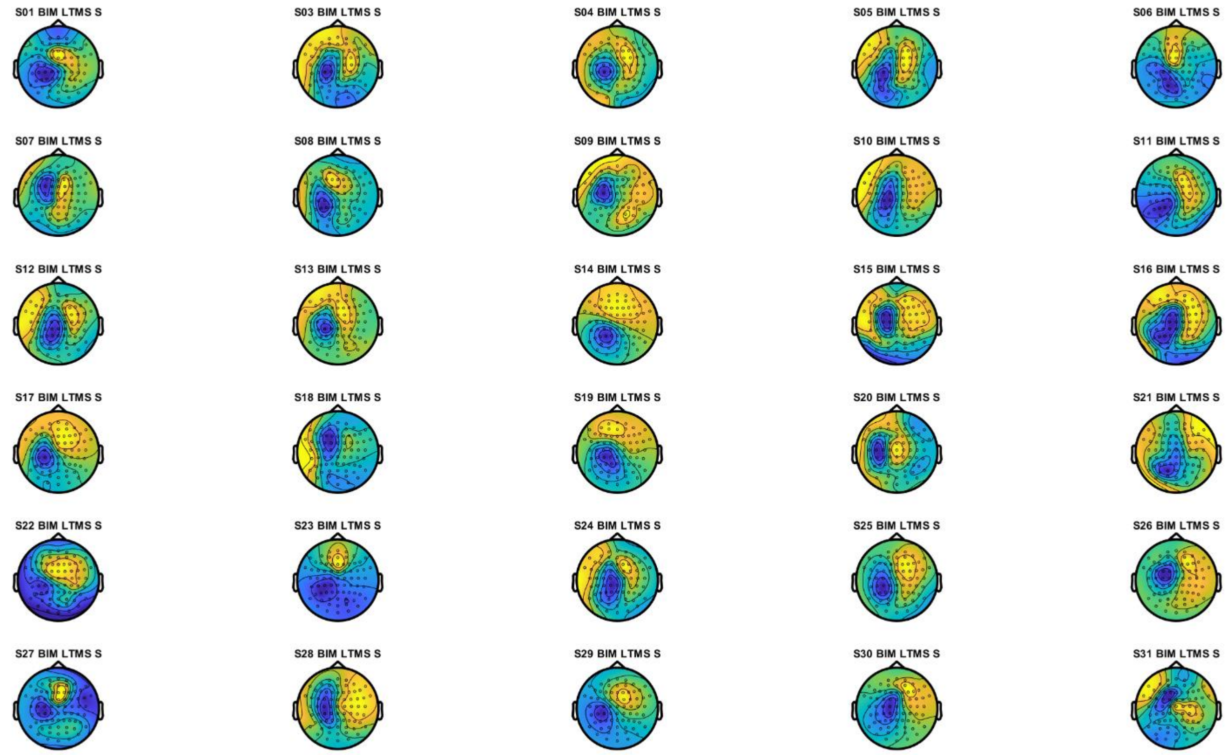
S01 BIM LTMS S
S03 BIM LTMS S
S04 BIM LTMS S
S05 BIM LTMS S
S06 BIM LTMS S
S07 BIM LTMS S
S08 BIM LTMS S
S09 BIM LTMS S
S10 BIM LTMS S
S11 BIM LTMS S
S12 BIM LTMS S
S13 BIM LTMS S
S14 BIM LTMS S
S15 BIM LTMS S
S16 BIM LTMS S
S17 BIM LTMS S
S18 BIM LTMS S
S19 BIM LTMS S
S20 BIM LTMS S
S21 BIM LTMS S
S22 BIM LTMS S
S23 BIM LTMS S
S24 BIM LTMS S
S25 BIM LTMS S
S26 BIM LTMS S
S27 BIM LTMS S
S28 BIM LTMS S
S29 BIM LTMS S
S30 BIM LTMS S
S31 BIM LTMS S

B)

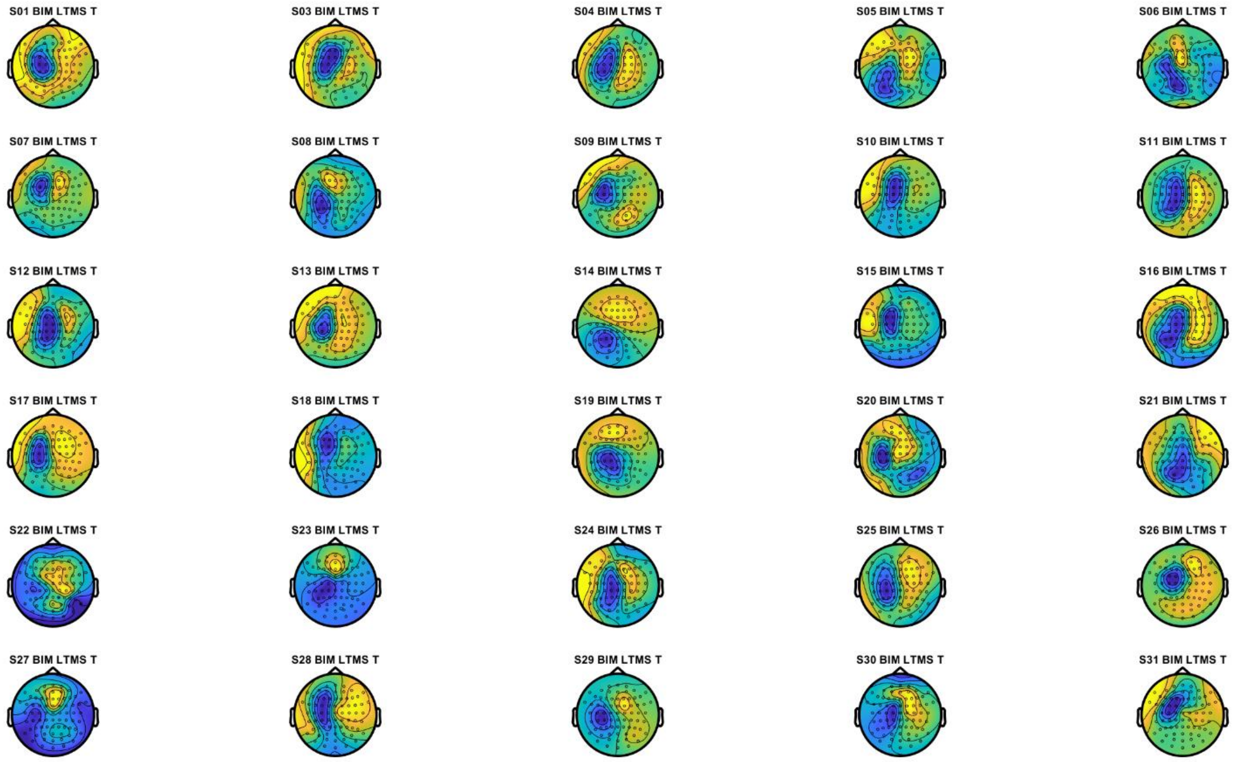
S01 BIM LTMS T
S03 BIM LTMS T
S04 BIM LTMS T
S05 BIM LTMS T
S06 BIM LTMS T
S07 BIM LTMS T
S08 BIM LTMS T
S09 BIM LTMS T
S10 BIM LTMS T
S11 BIM LTMS T
S12 BIM LTMS T
S13 BIM LTMS T
S14 BIM LTMS T
S15 BIM LTMS T
S16 BIM LTMS T
S17 BIM LTMS T
S18 BIM LTMS T
S19 BIM LTMS T
S20 BIM LTMS T
S21 BIM LTMS T
S22 BIM LTMS T
S23 BIM LTMS T
S24 BIM LTMS T
S25 BIM LTMS T
S26 BIM LTMS T
S27 BIM LTMS T
S28 BIM LTMS T
S29 BIM LTMS T
S30 BIM LTMS T
S31 BIM LTMS T

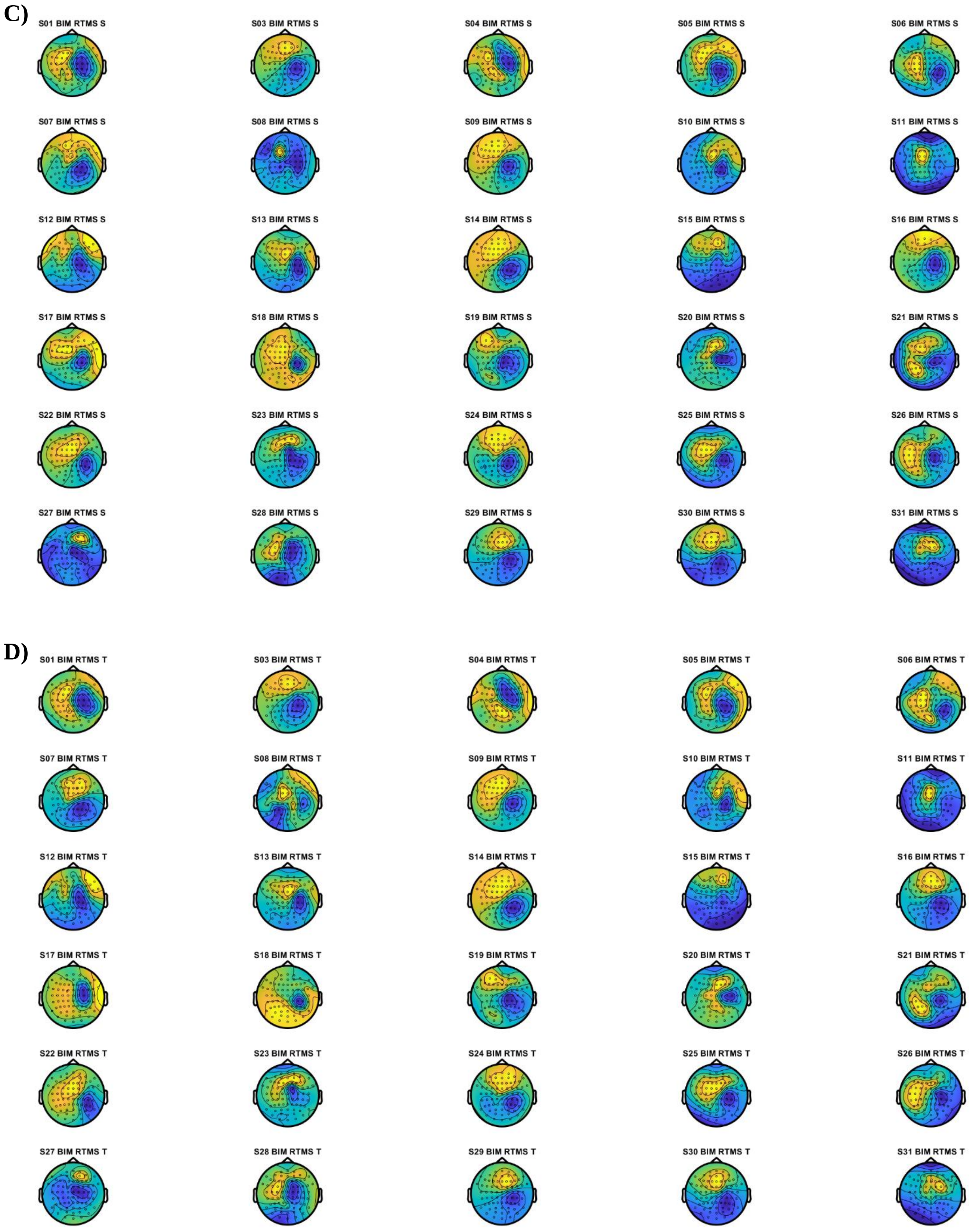

Fig. S4 M1-P15 topographies of individual subjects during the bimanual tasks. Plots were obtained on the average over a time-window of ± 5 ms around the individual peak, in the four experimental conditions: **A)** LTMS-Sequence; **B)** LTMS-Tapping; **C)** RTMS-Sequence; **D)** RTMS-Tapping.

Fig. S5

A)

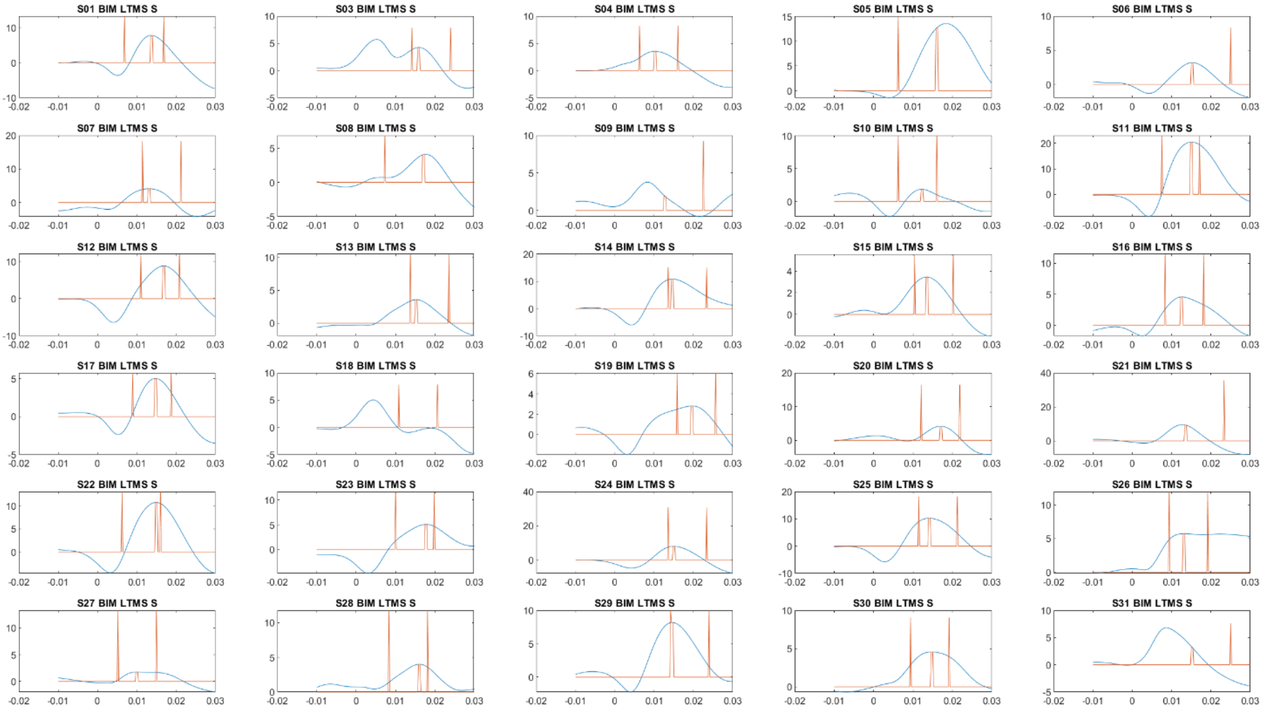
S01 BIM LTMS S
S03 BIM LTMS S
S04 BIM LTMS S
S05 BIM LTMS S
S06 BIM LTMS S
S07 BIM LTMS S
S08 BIM LTMS S
S09 BIM LTMS S
S10 BIM LTMS S
S11 BIM LTMS S
S12 BIM LTMS S
S13 BIM LTMS S
S14 BIM LTMS S
S15 BIM LTMS S
S16 BIM LTMS S
S17 BIM LTMS S
S18 BIM LTMS S
S19 BIM LTMS S
S20 BIM LTMS S
S21 BIM LTMS S
S22 BIM LTMS S
S23 BIM LTMS S
S24 BIM LTMS S
S25 BIM LTMS S
S26 BIM LTMS S
S27 BIM LTMS S
S28 BIM LTMS S
S29 BIM LTMS S
S30 BIM LTMS S
S31 BIM LTMS S

B)

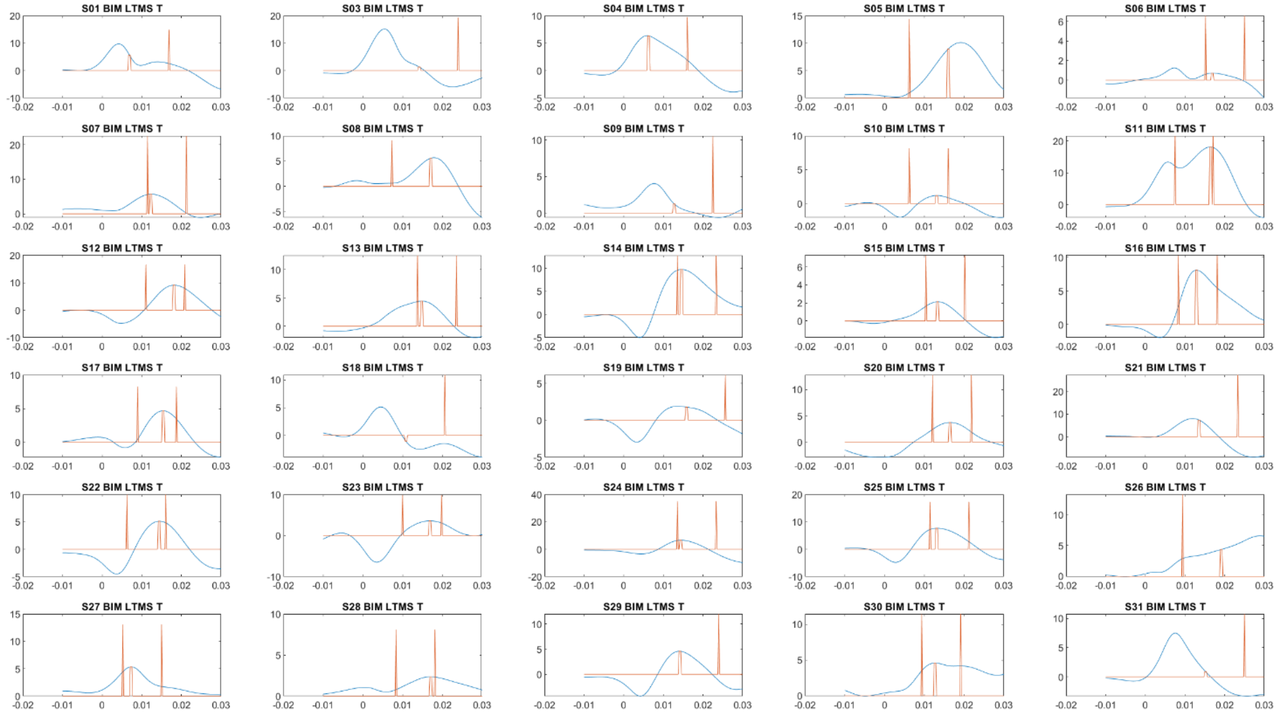
S01 BIM LTMS T
S03 BIM LTMS T
S04 BIM LTMS T
S05 BIM LTMS T
S06 BIM LTMS T
S07 BIM LTMS T
S08 BIM LTMS T
S09 BIM LTMS T
S10 BIM LTMS T
S11 BIM LTMS T
S12 BIM LTMS T
S13 BIM LTMS T
S14 BIM LTMS T
S15 BIM LTMS T
S16 BIM LTMS T
S17 BIM LTMS T
S18 BIM LTMS T
S19 BIM LTMS T
S20 BIM LTMS T
S21 BIM LTMS T
S22 BIM LTMS T
S23 BIM LTMS T
S24 BIM LTMS T
S25 BIM LTMS T
S26 BIM LTMS T
S27 BIM LTMS T
S28 BIM LTMS T
S29 BIM LTMS T
S30 BIM LTMS T
S31 BIM LTMS T

C)

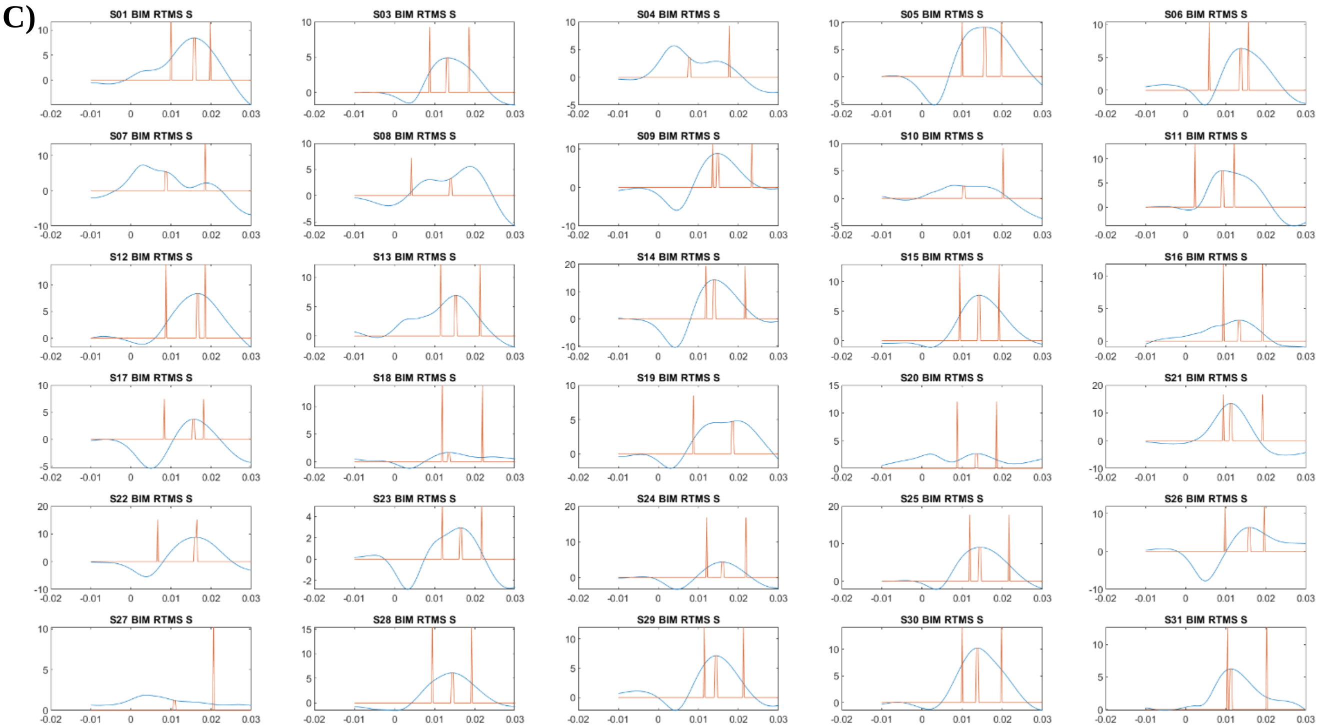

D)

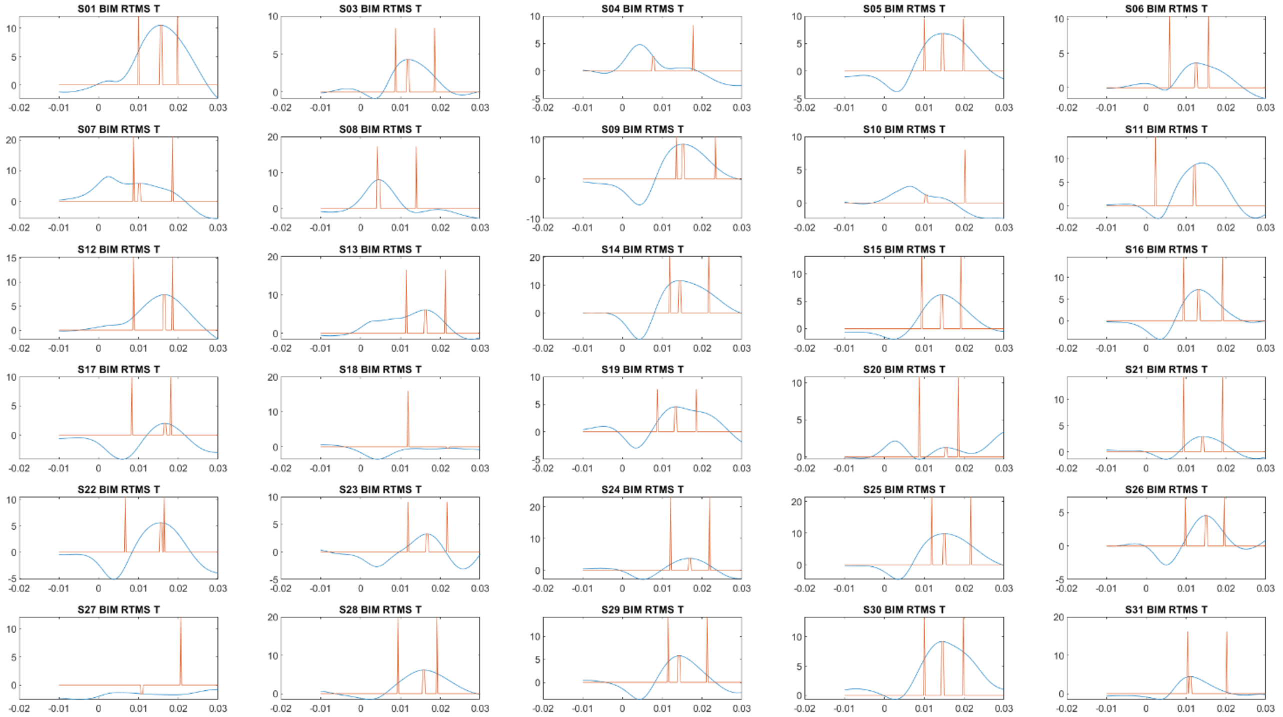

Fig S5. M1-P15 peaks of individual subjects during the bimanual tasks. The blue trace represents the EEG signal averaged over F4 and FC4 channels for LTMS, and over F3 and FC3 channels for RTMS, in the four experimental conditions: **A)** LTMS-Sequence; **B)** LTMS-Tapping; **C)** RTMS-Sequence; **D)** RTMS-Tapping. Orange traces represent the boundaries of individual time windows for peak identification and the peak.

Fig. S6

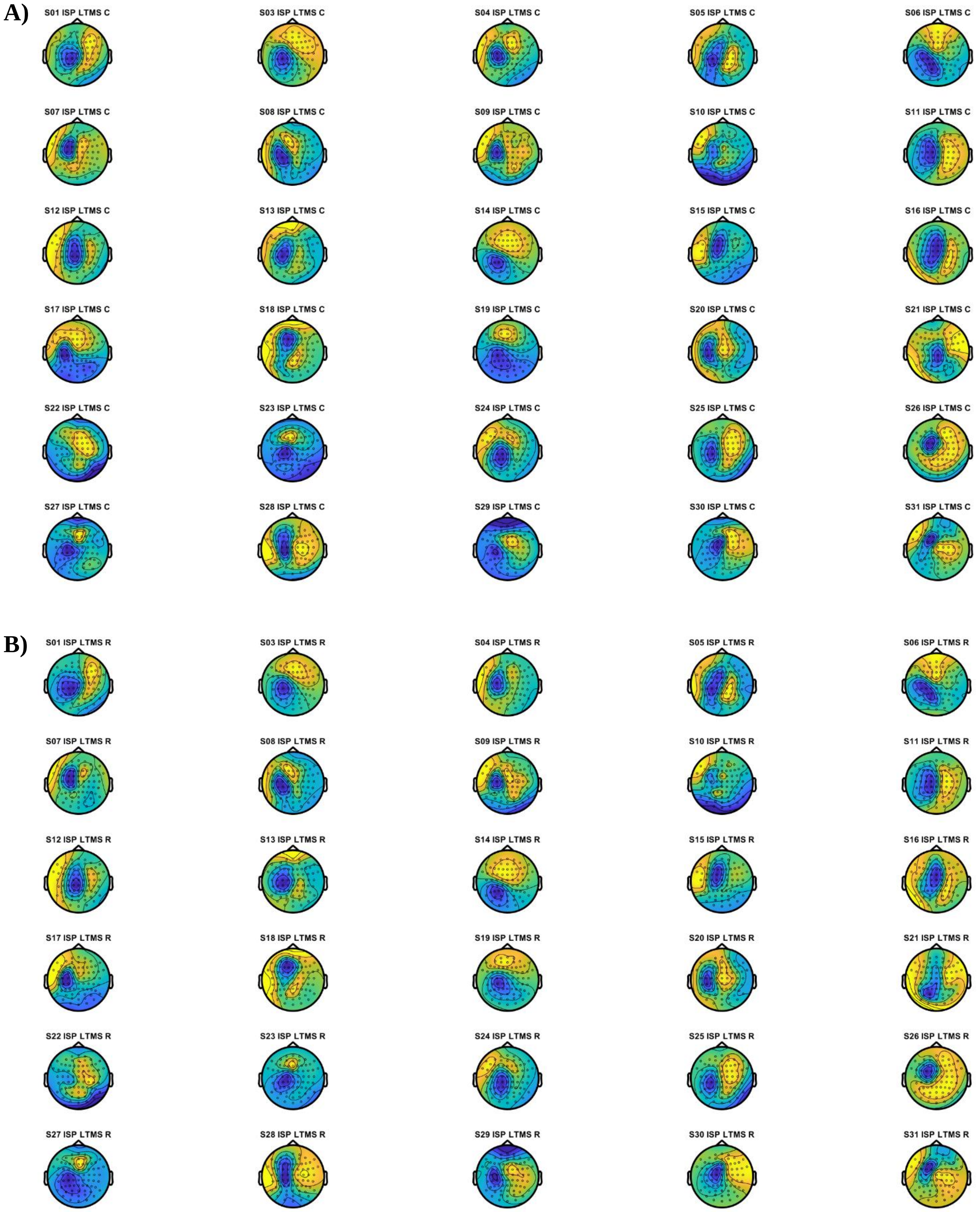
A)
S01 ISP LTMS C
S03 ISP LTMS C
S04 ISP LTMS C
S05 ISP LTMS C
S06 ISP LTMS C
S07 ISP LTMS C
S08 ISP LTMS C
S09 ISP LTMS C
S10 ISP LTMS C
S11 ISP LTMS C
S12 ISP LTMS C
S13 ISP LTMS C
S14 ISP LTMS C
S15 ISP LTMS C
S16 ISP LTMS C
S17 ISP LTMS C
S18 ISP LTMS C
S19 ISP LTMS C
S20 ISP LTMS C
S21 ISP LTMS C
S22 ISP LTMS C
S23 ISP LTMS C
S24 ISP LTMS C
S25 ISP LTMS C
S26 ISP LTMS C
S27 ISP LTMS C
S28 ISP LTMS C
S29 ISP LTMS C
S30 ISP LTMS C
S31 ISP LTMS C
B)
S01 ISP LTMS R
S03 ISP LTMS R
S04 ISP LTMS R
S05 ISP LTMS R
S06 ISP LTMS R
S07 ISP LTMS R
S08 ISP LTMS R
S09 ISP LTMS R
S10 ISP LTMS R
S11 ISP LTMS R
S12 ISP LTMS R
S13 ISP LTMS R
S14 ISP LTMS R
S15 ISP LTMS R
S16 ISP LTMS R
S17 ISP LTMS R
S18 ISP LTMS R
S19 ISP LTMS R
S20 ISP LTMS R
S21 ISP LTMS R
S22 ISP LTMS R
S23 ISP LTMS R
S24 ISP LTMS R
S25 ISP LTMS R
S26 ISP LTMS R
S27 ISP LTMS R
S28 ISP LTMS R
S29 ISP LTMS R
S30 ISP LTMS R
S31 ISP LTMS R

C)

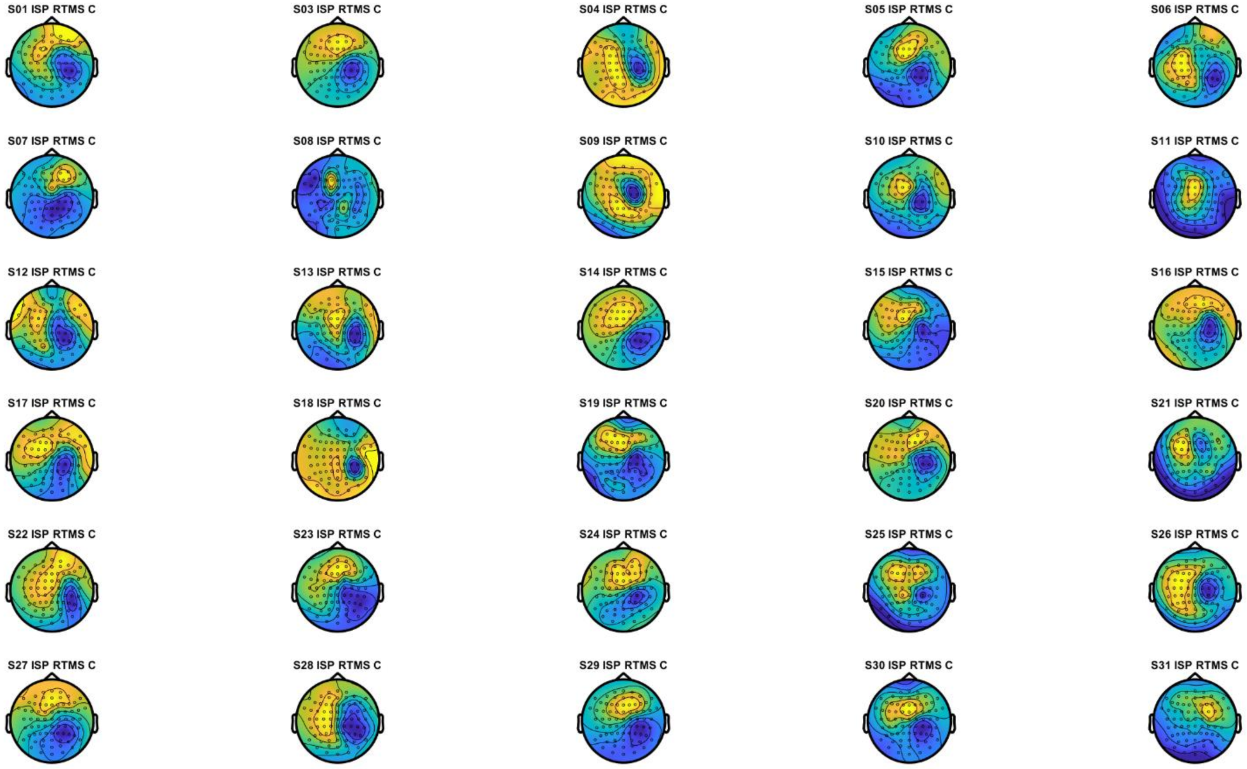

D)

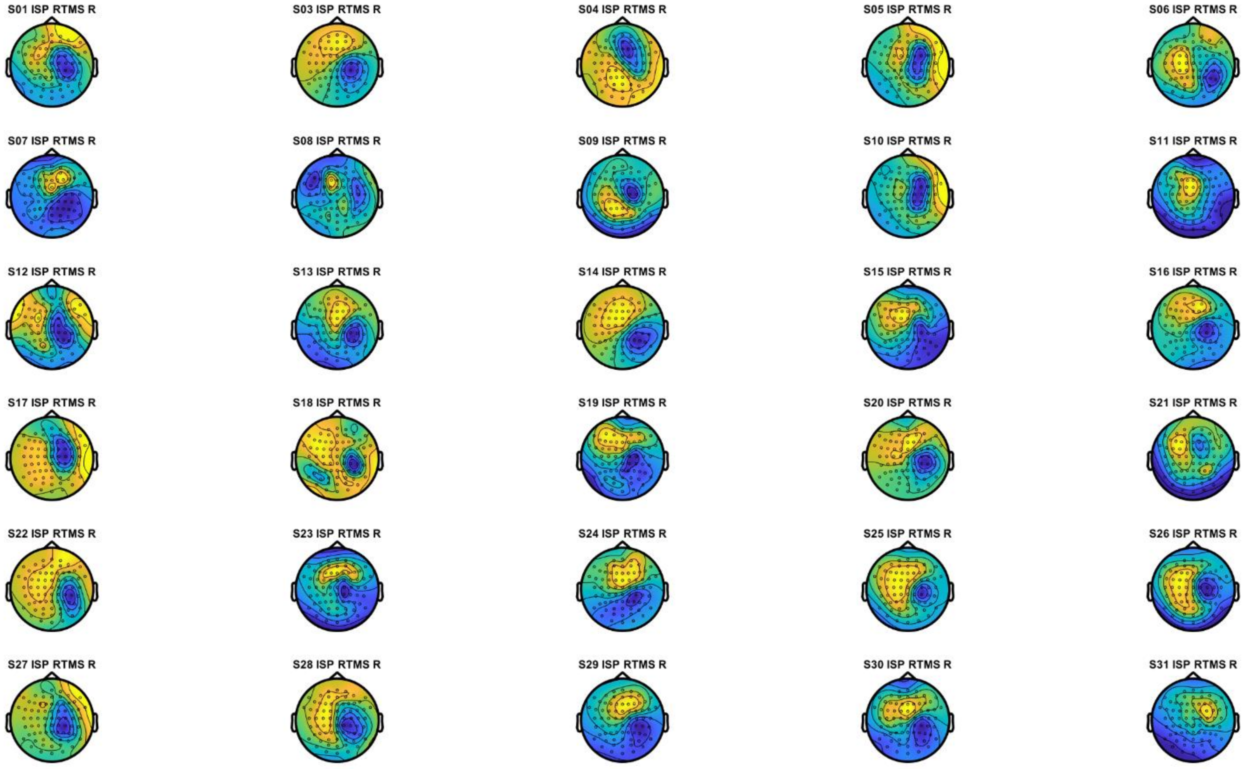

Fig. S6 M1-P15 topographies of individual subjects during the iSP paradigm. Plots were obtained on the average over a time-window of ± 5 ms around the individual peak, in the four experimental conditions: **A)** LTMS-Contracted; **B)** LTMS-Relaxed; **C)** RTMS-Contracted; **D)** RTMS-Relaxed.

Fig. S7

A)

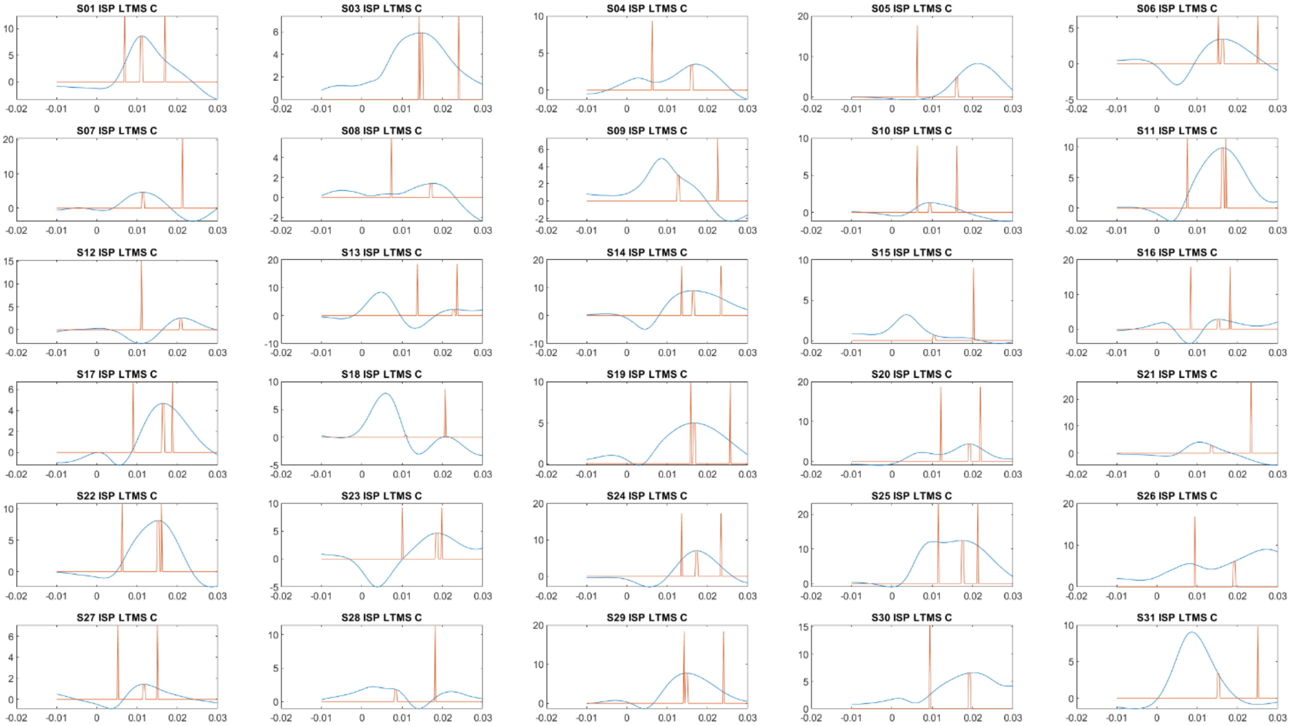
S01 ISP LTMS C
S03 ISP LTMS C
S04 ISP LTMS C
S05 ISP LTMS C
S06 ISP LTMS C
S07 ISP LTMS C
S08 ISP LTMS C
S09 ISP LTMS C
S10 ISP LTMS C
S11 ISP LTMS C
S12 ISP LTMS C
S13 ISP LTMS C
S14 ISP LTMS C
S15 ISP LTMS C
S16 ISP LTMS C
S17 ISP LTMS C
S18 ISP LTMS C
S19 ISP LTMS C
S20 ISP LTMS C
S21 ISP LTMS C
S22 ISP LTMS C
S23 ISP LTMS C
S24 ISP LTMS C
S25 ISP LTMS C
S26 ISP LTMS C
S27 ISP LTMS C
S28 ISP LTMS C
S29 ISP LTMS C
S30 ISP LTMS C
S31 ISP LTMS C

B)

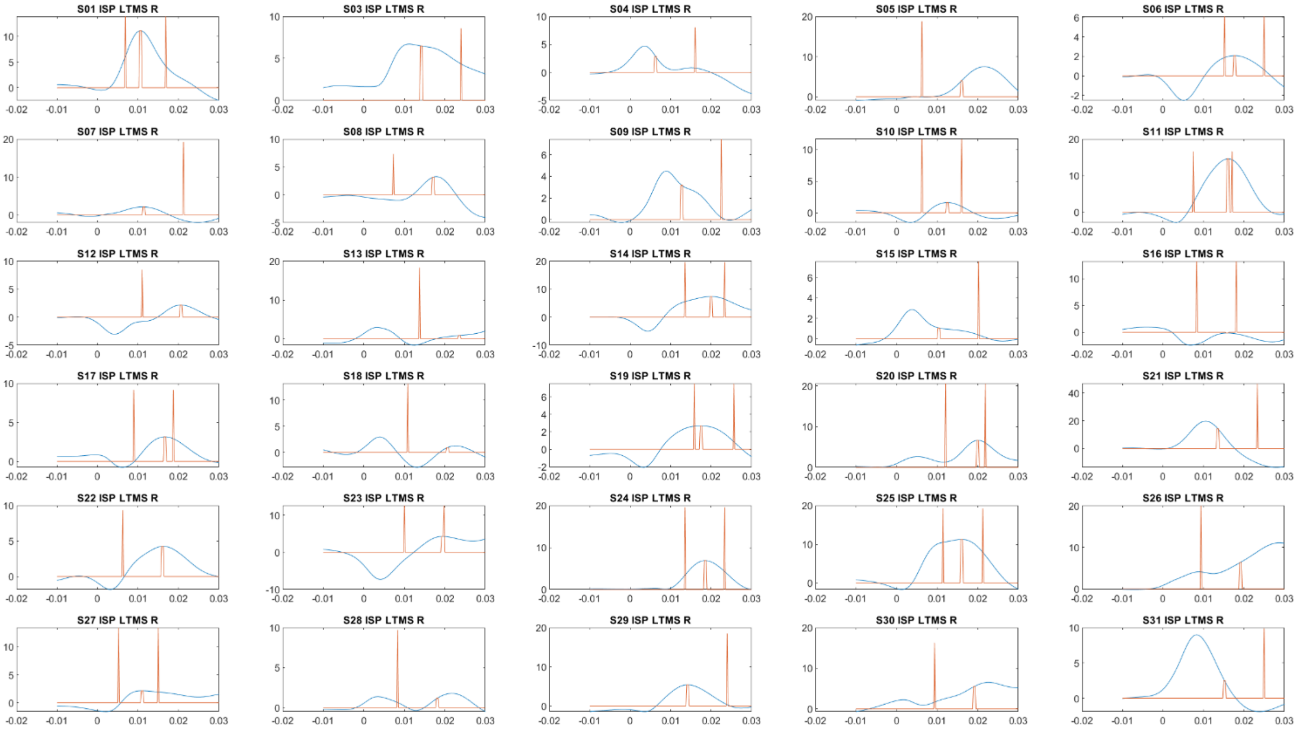
S01 ISP LTMS R
S03 ISP LTMS R
S04 ISP LTMS R
S05 ISP LTMS R
S06 ISP LTMS R
S07 ISP LTMS R
S08 ISP LTMS R
S09 ISP LTMS R
S10 ISP LTMS R
S11 ISP LTMS R
S12 ISP LTMS R
S13 ISP LTMS R
S14 ISP LTMS R
S15 ISP LTMS R
S16 ISP LTMS R
S17 ISP LTMS R
S18 ISP LTMS R
S19 ISP LTMS R
S20 ISP LTMS R
S21 ISP LTMS R
S22 ISP LTMS R
S23 ISP LTMS R
S24 ISP LTMS R
S25 ISP LTMS R
S26 ISP LTMS R
S27 ISP LTMS R
S28 ISP LTMS R
S29 ISP LTMS R
S30 ISP LTMS R
S31 ISP LTMS R

C)

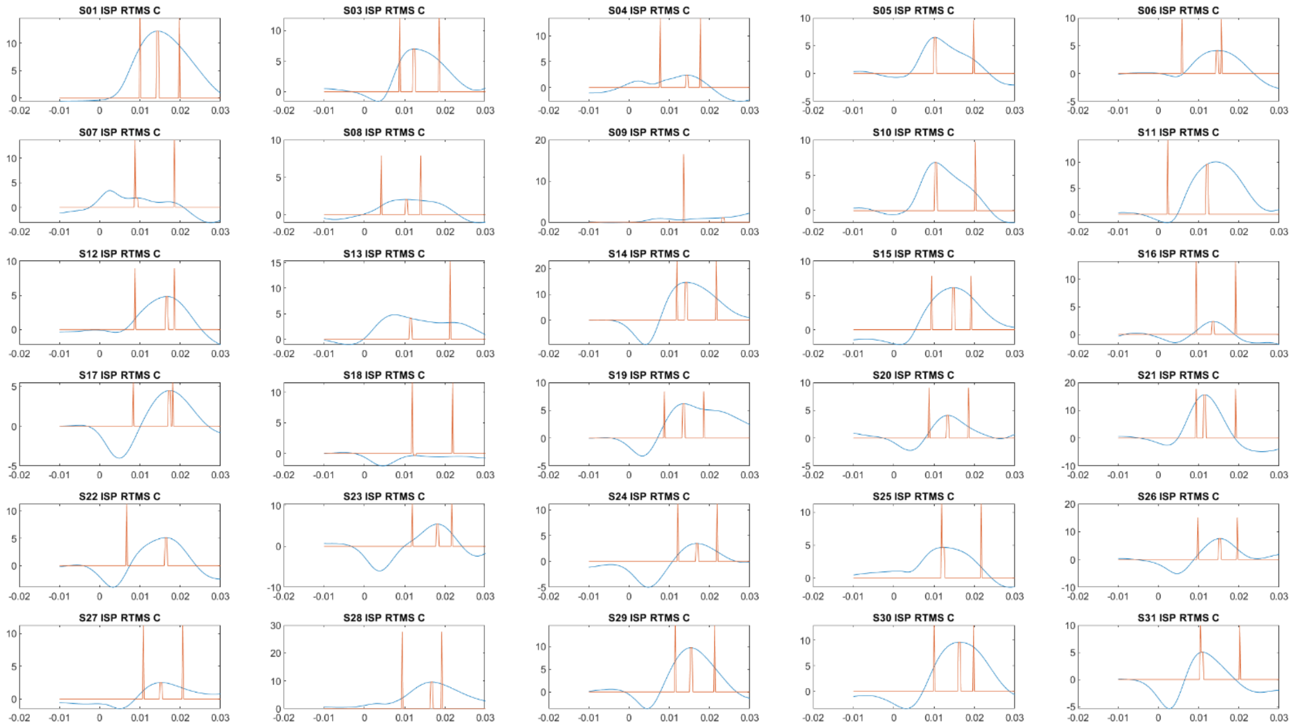

D)

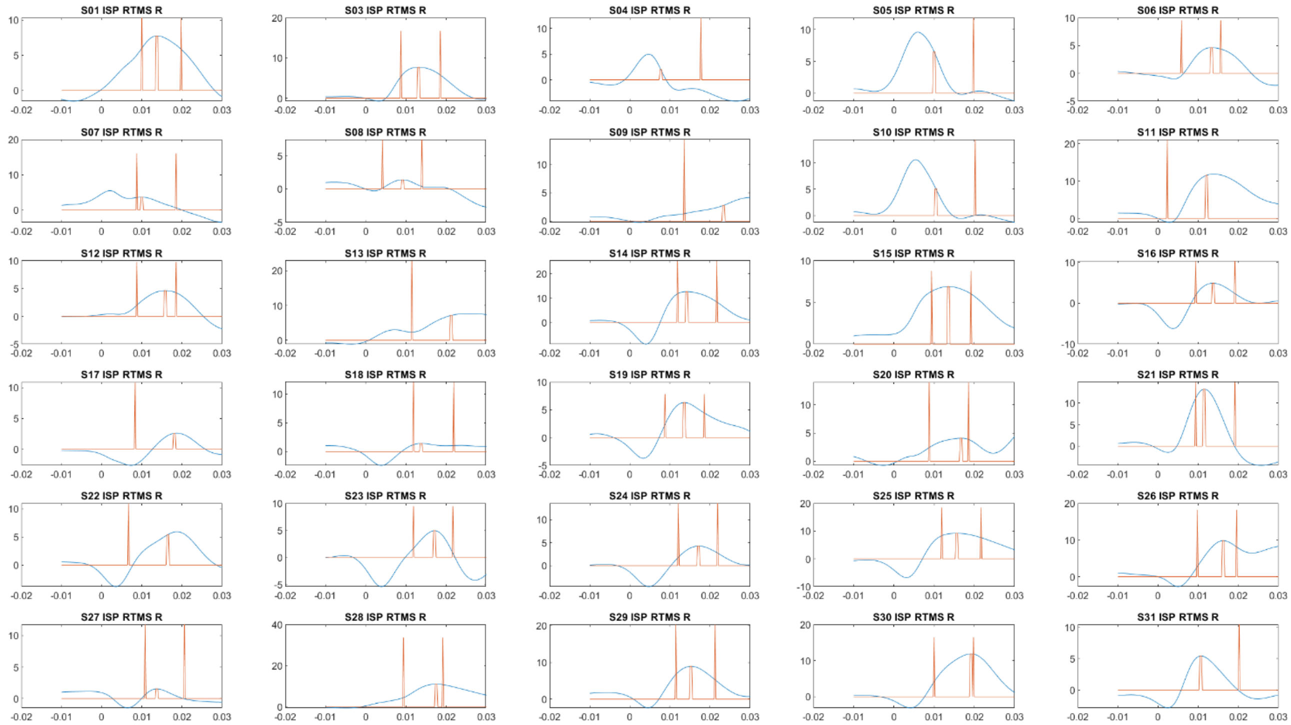

Fig S7. M1-P15 peaks of individual subjects during the iSP paradigm. The blue trace represents the EEG signal averaged over F4 and FC4 channels for LTMS, and over F3 and FC3 channels for RTMS, in the four experimental conditions: **A)** LTMS-Contracted; **B)** LTMS-Relaxed; **C)** RTMS-Contracted; **D)** RTMS-Relaxed. Orange traces represent the boundaries of individual time windows for peak identification and the peak.

Tables

Dependent variable	Independent variable	Fixed factor repeated within Subjects	β	*t*	*p*
i) M1-P15 amplitude as a measure of transcallosal inhibition					
Normalized iSP area	M1-P15 amplitude	Condition (4 measures per subject: *LTMS-Contracted, LTMS-Relaxed, RTMS-Contracted, RTMS-Relaxed*)	0.92	3.3	0.001 *
Normalized iSP area	Mean rMT		0.36	0.83	0.42
M1-P15 amplitude	Mean rMT		0.61	1.26	0.22
ii) Behavioral relevance of M1-P15 latency					
Inter-hand interval (*Sequence*)	LTMS M1-P15 latency in iSP paradigm	Tap (265 measures per subject)	0.03	0.74	0.47
Inter-hand interval (*Sequence*)	RTMS M1-P15 latency in iSP paradigm	Tap (265 measures per subject)	0.06	1.9	0.07
Inter-hand interval (*Sequence*)	LTMS/RTMS M1-P15 latency in iSP paradigm	Tap (265 measures per subject)	-0.2	-0.42	0.69
Inter-hand interval (*Sequence*)	LTMS M1-P15 latency in bimanual *Sequence*	Tap (265 measures per subject)	-0.004	-0.09	0.93
Inter-hand interval (*Sequence*)	RTMS M1-P15 latency in bimanual *Sequence*	Tap (265 measures per subject)	0.027	0.74	0.48
Inter-hand interval (*Sequence*)	LTMS/RTMS M1-P15 latency in bimanual *Sequence*	Tap (265 measures per subject)	-0.028	-0.76	0.46

Table S1. Structure and results of LMMs. All LMMs models were run with random slopes and intercepts, and with Subjects as random factor. *β, t* and *p* values refer to the independent variable. Asterisks indicates significant effects ($p < 0.05$).

Dependent variable	Factor (levels)	F	Degrees of freedom	*p*
ii) Behavioral relevance of M1-P15 latency				
Mean inter-hand interval *(Sequence)*	Block *(Block 1, Block2, Block3)*	0.75	2, 58	0.48
M1-P15 latency in bimanual tasks	Hemisphere *(LTMS, RTMS)*	0.02	1, 29	0.9
	Task *(Sequence, Tapping)*	0.56	1, 29	0.46
	Hemisphere * Task	1.91	1, 29	0.18
M1-P15 latency in iSP paradigm	Hemisphere *(LTMS, RTMS)*	2.13	1, 28	0.16
	Contraction *(Contracted, Relaxed)*	0.47	1, 28	0.5
	Hemisphere * Contraction	0.01	1, 28	0.92
iii) Modulation of interhemispheric inhibition				
ISP normalized area	Hemisphere *(LTMS, RTMS)*	4.24	1, 28	0.049 *
	Contraction *(Contracted, Relaxed)*	1.05	1, 28	0.31
	Hemisphere * Contraction	0.95	1, 28	0.34
M1-P15 amplitude in iSP paradigm	Hemisphere *(LTMS, RMTS)*	3.69	1, 28	0.065
	Contraction *(Contracted, Relaxed)*	2.13	1, 28	0.16
	Hemisphere * Contraction	0.29	1, 28	0.6
M1-P15 amplitude in bimanual tasks	Hemisphere (LTMS, RMTS)	0.03	1, 29	0.87
	Task *(Sequence, Tapping)*	6.43	1, 29	0.017 *
	Hemisphere * Task	1.79	1, 29	0.19

Table S2. Structure and results of rm-ANOVAs. Please note that the statistical analyses on the data collected during the bimanual tasks were performed on 30 subjects, while the ones during the iSP paradigm were performed on 29 subjects, because of the exclusion of 1 subject from iSP analysis. Asterisks indicates significant effects ($p < 0.05$).

Dependent variables	Factor (levels)	MATS	Degrees of freedom	*p*
iii) Modulation of interhemispheric inhibition				
ISP normalized area<br>M1-P15 amplitude in ISP paradigm	Hemisphere *(LTMS, RTMS)*	6.66	2	0.023 *
	Contraction *(Contracted, Relaxed)*	0.64	2	0.22
	Hemisphere * Contraction	1	2	0.53

Table S3. Structure and results of rm-MANOVA. Considering the 2x2 design of the rm-MANOVA, a semi-parametric model was performed using the MANOVA.RM package in R (10000 iterations, modified ANOVA-type statistic – MATS, parametric bootstrap approach for resampling). Post-hoc analyses were performed by computing separate 2 X 2 rm-ANOVAs on M1-P15 amplitude and iSP normalized area, which can be found in Table 2. Asterisks indicates significant effects ($p < 0.05$).
